# Discovering Genetic Factors for psoriasis through exhaustively searching for significant second order SNP-SNP interactions

**DOI:** 10.1038/s41598-018-33493-w

**Published:** 2018-10-12

**Authors:** Kwan-Yeung Lee, Kwong-Sak Leung, Nelson L. S. Tang, Man-Hon Wong

**Affiliations:** 1 Department of Computer Science and Engineering, the Chinese University of Hong Kong, Hong Kong, China; 2 Department of Chemical Pathology, the Chinese University of Hong Kong, Hong Kong, China

## Abstract

In this paper, we aim at discovering genetic factors of psoriasis through searching for statistically significant SNP-SNP interactions exhaustively from two real psoriasis genome-wide association study datasets (phs000019.v1.p1 and phs000982.v1.p1) downloaded from the database of Genotypes and Phenotypes. To deal with the enormous search space, our search algorithm is accelerated with eight biological plausible interaction patterns and a pre-computed look-up table. After our search, we have discovered several SNPs having a stronger association to psoriasis when they are in combination with another SNP and these combinations may be non-linear interactions. Among the top 20 SNP-SNP interactions being found in terms of pairwise p-value and improvement metric value, we have discovered 27 novel potential psoriasis-associated SNPs where most of them are reported to be eQTLs of a number of known psoriasis-associated genes. On the other hand, we have inferred a gene network after selecting the top 10000 SNP-SNP interactions in terms of improvement metric value and we have discovered a novel long distance interaction between *XXbac-BPG154L12.4* and *RNU6-283P* which is not a long distance haplotype and may be a new discovery. Finally, our experiments with the synthetic datasets have shown that our pre-computed look-up table technique can significantly speed up the search process.

## Introduction

Psoriasis is a common polygenic chronic inflammatory skin disease affecting up to 3% of population^1^. Recently, genome-wide association study (GWAS) provided the first opportunity to have a comprehensive screen for susceptibility genes and up to 50 loci had been reported^1,2^. The discovery of novel susceptibility genes by GWAS included genes coding for key cytokines involved in Th17 activation (like *IL-12B*, *IL23A* and *IL23R*) and *NF-κB* pathway also contributed to the aetiology (*TNFAIP3* and *TNIP1*)^3^. In addition, macrophage and dendritic cells are also involved. *HLA-C* locus had been a known susceptibility gene before the GWAS era^4^. Exaggerated expression of keratinocyte antigens (like *LCE3D*) was also identified as susceptibility genes^5^.

Although many predisposition genes for psoriasis have been identified, individually they accounted for very small effect size, for example their odds ratios were typically less than 1.2^6^. While heritability of psoriasis had been estimated to be as high as 60–90%, the genetic risk of all variants added up could only account for one-fourth of susceptibility due to genetics. This phenomenon is called missing heritability^7^. One possible source of unaccounted risk is interactions (including gene-gene and gene-environment interaction), which is the risk of certain genotype may be altered to a large extent in the presence of another risk factor, which is also known as non-additive effects^6,8,9^. Various algorithms have been proposed to detect the interactions between SNPs or genes in GWAS data^10,11^. They all faced the difficulties of large search space, exponential increase in SNP combination with increasing level of interaction, and limited statistical basis of the proposed methods. On the other hand, examples of epistasis were found in model organisms and human diseases^9,12,13^. We had proposed a biological framework of gene-gene interaction and suggested that typical samples size (thousands of cases and controls) should have sufficient power to detect such gene-gene interaction with simulation data^14^. There were also some suggestions of epistasis in psoriasis but replication in subsequent studies were lacking^3,8^.

Biological pathways are regulated by the interactions among bio-molecules constructed according to the genetic instructions stored in deoxyribonucleic acid (DNA). A single nucleotide polymorphism (SNP) is a variation at a specific DNA position among a population of organisms which may affect the structure of these bio-molecules. In a genome-wise association study (GWAS), DNA sequences of a large population of patient samples (cases) of a particular genetic disease and healthy samples (controls) are collected and researchers can discover disease-associated SNPs through comparing the DNA sequences between cases and controls^15^. These DNA sequences can be arranged into a matrix *A* where each column (except the last column) corresponds to a SNP and each row corresponds to a sample as shown in Table 1. Each entry *A*_*i,j*_ corresponds to the genotype of *i*^*th*^ sample at *j*^*th*^ SNP under the encoding scheme shown in Table 2. Each sample is either labelled as ‘case’ or ‘control’ through the value of the last column of the matrix. A traditional approach for finding statistically significant SNPs which has been widely adapted in many GWAS researches is to perform statistical tests after building a contingency table for each column of matrix *A*.

**Table 1:** This table shows an example of a GWAS dataset.

	SNP_1_	SNP_2_	${\boldsymbol{\cdots }}$	SNP_*m*_	Status
Sample_1_	1	1	$\cdots$	1	T
Sample_2_	2	1	$\cdots$	2	F
$\vdots$	$\vdots$	$\vdots$	$\ddots$	$\vdots$	$\vdots$
Sample_*n*-1_	3	2	$\cdots$	2	F
Sample_*n*_	3	2	$\cdots$	2	F

**Table 2:** This table shows the encoding scheme for SNP genotype.

Original Genotype	Encode Value
Missing Data	0
Major Allele, Major Allele	1
Major Allele, Minor Allele	2
Minor Allele, Minor Allele	3

**Figure 1:**
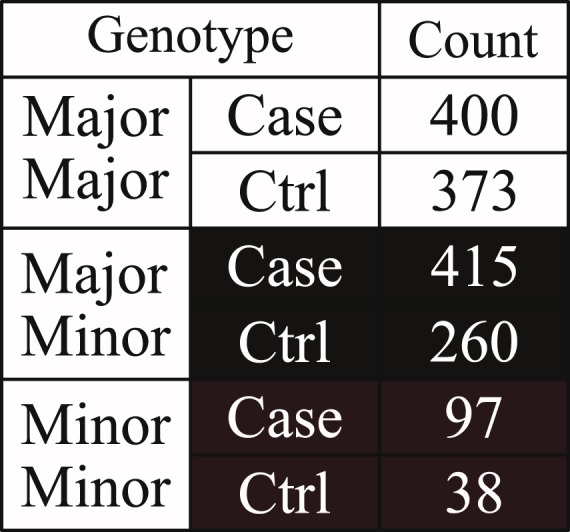
In this figure, the table shows the distribution of case and control under different genotypes of SNP rs3130048 in dataset phs000019.v1.p1. The 1 d.f. chi-square *p*-value and odds ratio of rs3130048 are 3.96 × 10^−6^ and 1.6021 respectively.

**Figure 2:**
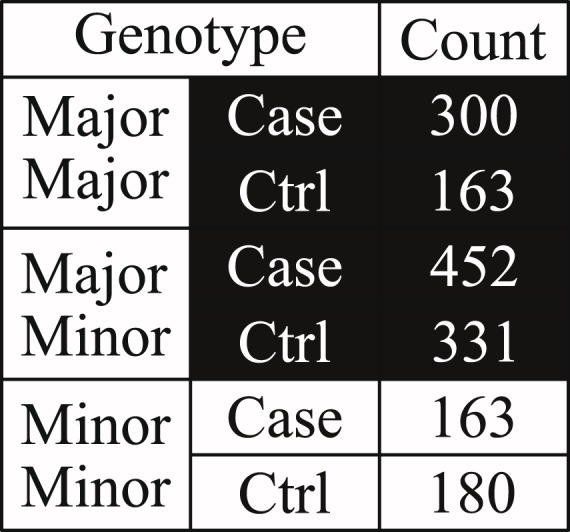
In this figure, the table shows the distribution of case and control under different genotypes of SNP rs3132486 in dataset phs000019.v1.p1. The 1 d.f. chi-square *p*-value and odds ratio of rs3132486 are 2.06 × 10^−5^ and 1.6810 respectively.

**Figure 3:**
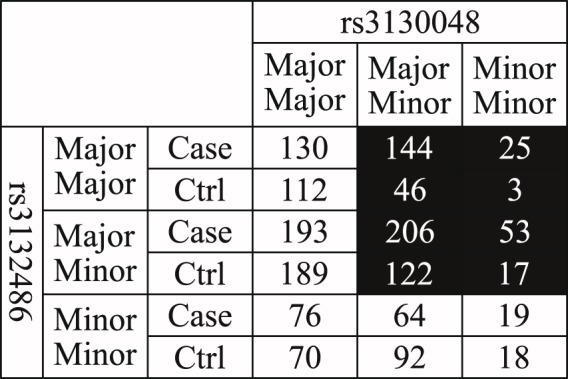
In this figure, the table shows the distribution of case and control under genotype of the combination of SNP rs3132486 and rs3130048 in dataset phs000019.v1.p1. The 1 d.f. chi-square *p*-value and odds ratio of the combination of rs3132486 and rs3130048 are 2.52 × 10^−14^ and 2.2719 respectively.

As previously discussed, the cause of many genetic diseases can be better explained through certain combinations of SNPs (i.e. SNP-SNP interactions) rather than a number of independent SNPs alone^16,17^. Although many SNPs are weakly associated to the genetic diseases when they are analysed independently, some of them may show a stronger association only when they are analysed in combination with other SNPs. A typical example of this phenomenon is shown in Figs 1–3 and Supplementary Fig. S1. In Figs 1 and 2, rs3132486 and rs3130048 from dataset phs000019.v1.p1 are both weakly associated to psoriasis with a 1 degree of freedom (d.f.) chi-square *p*-value less significant than 1 × 10^−6^ and an odds ratio smaller than 1.7 if they are analysed independently. Meanwhile in Fig. 3, the combination of rs3132486 and rs3130048 are significantly associated to psoriasis with a 1 d.f. chi-square *p*-value of 2.52 × 10^−14^ and an odds ratio of 2.2719. Therefore, rs3132486 and rs3130048 may have a potential non-linear interaction associated to psoriasis. Similar but much weaker phenomena have also been observed between SNPs among two known psoriasis associated gene-gene interactions (*HLA-C*, *IL12B*)^8^ and (*HLA-C*, *TNFAIP3*)^3^ and are shown in Supplementary Figs S2 and S3 respectively.

In previous GWAS researches, filtering out a large proportion of statistically insignificant SNPs or performing search with greedy and stochastic search algorithms are two popular techniques for shrinking the search space of SNP-SNP interactions^18,19^. Any researcher who performs his/her analysis with a search space confined to statistically significant single SNPs only will prematurely filtered out some disease-associated SNPs which are statistically insignificant on their own similar to the ones shown in Figs 1 and 2. Meanwhile, greedy and stochastic search selectively explore the search space under the guidance of heuristic functions thus they may also fail to detect some disease-associated but statistically insignificant SNPs as well. However, performing exhaustive search for interactions among *k* different SNPs (i.e. *k*^*th*^ order SNP-SNP interaction) would take a time complexity of *O*(*m*^*k*^*n*) where *m* is the number of SNPs and *n* is the number of samples. Given the fact that there are around 38 million SNPs with one million tag SNPs across the whole human genome^20^ and a GWAS dataset often contains thousands of samples and hundreds of thousands of SNPs, this problem is difficult to be solved without any advanced computational technique. Driven by the continuous growth of computational power, it is now becoming possible to perform exhaustive search for 2^*nd*^ order SNP-SNP interactions in a GWAS dataset within a reasonable amount of time^21–28^. However, existing exhaustive search algorithms are not driven by any biological knowledge and they evaluate the disease association of each SNP combination solely based on heuristic or statistical parameters. Therefore, they may found statistical significant SNP-SNP interactions which may not be biologically interpretable.

We have developed an exhaustive search algorithm driven by eight biological plausible SNP-SNP interaction and applied it on two psoriasis GWAS datasets (phs000019.v1.p1 and phs000982.v1.p1). We have first discovered a number of statistically significant SNP-SNP interactions which may have a stronger association to psoriasis then their component SNPs measured independently similar to the ones shown in Fig. 3 where the SNPs involved in these interactions are far less significant when they are considered individually. After that, we have discovered 27 novel potential psoriasis-associated SNPs among the top 20 statistically significant SNP-SNP interactions in terms of *p*-value and improvement metric value. Most of these novel potential psoriasis-associated SNPs are reported to be Expression quantitative trait loci (eQTLs) of known psoriasis-associated genes like *HLA-B* and *HLA-C* in GTEx Portal^29^. After mapping the nearest gene to each SNP involved in top 10000 SNP-SNP interactions in terms of improvement metric value from both dataset, we have constructed a disease-associated gene network. In our network, almost half of the gene-gene interactions inferred are consistent with existing literature. Meanwhile, some of the remaining gene-gene interactions are potentially due to the long-distance haplotype interactions presence in the HLA region of Chromosome 6. Furthermore, we have discovered an interaction between 2 SNPs located in gene *XXbac-BPG154L12.4* and *RNU6-283P* of the HLA loci, which doesn’t correspond to any short-distance or even long-distance haplotype interactions and hasn’t been reported in existing literature as well. Therefore, the interaction between *XXbac-BPG154L12.4* and *RNU6-283P* is a potential new discovery. In addition, we have shown that counting contingency table through a pre-computed look-up table is effective in speeding up the process of exhaustive search.

## Results

### GWAS datasets

We have downloaded two psoriasis GWAS datasets namely phs000019.v1.p1 and phs000982.v1.p1 from the database of Genotypes and Phenotypes (dbGaP). Data pre-processing has been performed on these two datasets to remove low quality SNPs and samples with Plink^30^. The parameters of data cleansing can be found as Supplementary Tables S1 and S2 which followed the common recommendations from NCBI^31^.

After data cleansing, there are 352945 SNPs and 1593 samples (cases: 917, controls: 676) in dataset phs000019.v1.p1 and there are 790527 SNPs and 2689 samples (cases: 1363, controls: 1326) in dataset phs000982.v1.p1. The genotypes of every SNP in these two datasets are encoded as 0, 1, 2, 3 according to the encoding scheme shown in Table 2.

### Measurement metric for ranking and filtering SNP-SNP interaction

In this paper, SNP-SNP interactions are prioritised with the following two measurement metrics. The pairwise *p*-value of a 2^*nd*^ order SNP combination under a particular genotype interaction pattern is referring to the 1 d.f. chi-square *p*-value of its 2 × 2 contingency table founded from a real psoriasis dataset with the procedure illustrated in the Methods section. Additionally, we have defined another measurement metric called improvement metric value to prioritise statistically significant SNP-SNP interactions where its two components SNPs are far less associated to psoriasis when they are analysed independently. It compares the pairwise *p*-value of a 2^*nd*^ order SNP combination under a particular genotype interaction pattern against the standalone 1 d.f. chi-square *p*-value of both of its component SNPs and can be calculated by equation 1. If a 2^*nd*^ order SNP combination has a higher improvement metric value, its component SNPs are far more statistically significant when they are considered as a SNP combination rather than separately considered as two independent SNPs.

1 $$Improvement\_Metric\_Value=MIN(\frac{SNP1\,p \mbox{-} value}{{2}^{nd}\,Order\,SNP\,p \mbox{-} value},\,\frac{SNP2\,p \mbox{-} value}{{2}^{nd}\,Order\,SNP\,p \mbox{-} value})$$

### Exhaustive search on psoriasis datasets phs000019.v1.p1 and phs000982.v1.p1

We have performed exhaustive search on the two cleansed psoriasis GWAS datasets to discover biologically plausible and statistically significant 2^*nd*^ order SNP-SNP interactions. There are 62284910040 and 312466073601 unique 2^*nd*^ order SNP combinations in datasets phs000019.v1.p1 and phs000982.v1.p1 respectively. Each SNP combination is subjected to eight 1 d.f. chi-square statistic tests corresponding to eight genotype interaction patterns and eight pairwise *p*-value is thus calculated. Therefore, there are 498279280320 and 2499728588808 statistical tests performed on phs000019.v1.p1 and phs000982.v1.p1 datasets respectively. Among these tests, there are 3058119 and 59810682 statistically significant pairwise SNP-SNP interactions found in datasets phs000019.v1.p1 and phs000982.v1.p1 which have a pairwise *p*-value smaller than 1 × 10^−13^ and 1 × 10^−14^ respectively. We have sorted these statistically significant SNP-SNP interactions by their pairwise *p*-value and improvement metric value separately for further analysis.

### Analysis on top 20 most statistical significant SNP-SNP interaction

After sorting the interactions found in datasets phs000019.v1.p1 and phs000982.v1.p1 by their pairwise *p*-value, we have selected the top 20 statistical significant SNP-SNP interactions in terms of pairwise *p*-value which are listed in Supplementary Tables S3 and S4 respectively.

Among the interactions shown in Supplementary Table S3, there are six SNPs which are already reported to be associated to psoriasis in existing literature: rs12191877^3,32–35^, rs1265078^32,33,36^, rs2894207^32^, rs3130467^32^, rs3130517^32^ and rs3130573^33^. According to GTEx Portal^29^, there are nine SNPs acting as eQTLs of the following four known psoriasis associated genes, *HLA-C*^37,38^, *HCP5*^39,40^, *PSORS1C1*^41,42^ and *MICB*^43^. First, rs2244027 and rs2894176 are found to be eQTLs of *HLA-C*. After that, rs2516417, rs2516510, rs2523708 and rs2844502 are found to be eQTLs of *HCP5*. Then, rs9262492 and rs9262498 are found to be eQTLs of *PSORS1C1*. Finally, rs2534666 is found to be eQTLs of *MICB*. Therefore, these nine SNPs have a high potential to be associated to psoriasis and may be new discoveries.

On the other hand among the interactions we have selected in dataset phs000982.v1.p1, there are 3 psoriasis associated SNPs which are already reported in existing literature: rs13203895^44^, rs10484554^39,45–47^ and rs17728338^47–50^. Meanwhile, rs4349859 and rs4418214 have already been found to be strongly associated to *HLA-b27* allele^51^ and *HIV* infection^52^ respectively in other studies. According to GTEx Portal^29^, rs45533135 is an eQTLs of MICA. Since *HIV*^53^, *HLA-B*^37^ and *MICA*^54–56^ are strongly associated to psoriasis, these three SNPs have a high potential to be associated to psoriasis and may be new discoveries.

### Analysis on SNP-SNP interactions with the top 20 improvement metric value

After sorting the statistically significant SNP-SNP interactions found in both datasets by their improvement metric value, we have selected top 20 statistically significant SNP-SNP interactions in terms of improvement metric value and are listed in Supplementary Tables S5 and S6. We can observe that these SNP-SNP interactions have a pairwise *p*-value much smaller than the standalone *p*-value of their component SNPs. Therefore, these interactions may be non-linear and further verification through wet-lab experiments should be performed in the future.

In Supplementary Table S5, there are nine SNPs which are present in Supplementary Table S3. Meanwhile, there are six SNPs rs9380237, rs7756521, rs2853950, rs2844645, rs7773175 and rs8365 which are not found in Supplementary Table S3. Among these six SNPs, rs7773175^32^, rs9380237^32^ and rs2853950^57^ are literature reported psoriasis associated SNPs. According to GTEx Portal^29^, there are three SNPs which are eQTLs of the following two known psoriasis associated genes, *PSORS1C1*^41,42^ and *HLA-DQB1*^58,59^. First, rs7756521 and rs2844645 are found to be eQTLs of *PSORS1C1*. After that, rs8365 is found to be an eQTL of *HLA-DQB1*. The association between these three SNPs and psoriasis may be new discoveries.

Meanwhile there are no common SNPs between Supplementary Tables S6 and S4. Among the SNPs found in Supplementary Table S6, we observed that there is a SNP rs1576 which is reported to be associated to psoriasis in existing literature^60,61^. Meanwhile, SNPs rs1265112 and rs746647 are reported to be in complete linkage disequilibrium (*r*^2^ = 1.00) with the SNP rs1576 in an existing literature^62^. Therefore, SNPs rs1265112 and rs746647 can both be considered as a proxy SNP of a literature-reported psoriasis associated SNP and they are not new discoveries. According to GTEx Portal^29^, there are ten SNPs which are eQTLs of the following two known psoriasis associated genes, *HLA-C*^37,38^ and *MICA*^54–56^. Four of them are found to be reported as eQTLs of *HLA-C* namely rs2517985, rs1265079, rs1265114 and rs1265067. Meanwhile, six of them are found to be reported as eQTLs of *MICA* namely rs4358666, rs2395491, rs4624908, rs7754026, rs13194571 and rs7775117. The association between these ten eQTL SNPs and psoriasis may be new discoveries.

### Further analysis on the component SNPs of the SNP-SNP interactions being discovered with CADD SNP annotation

By referring to the genome assembly GRCh37 published by Genome Reference Consortium, the genomic position of every SNP can be retrieved. After knowing the genomic position of every SNP, we have annotated every component SNP of the top 20 SNP-SNP interactions in terms of improvement metric value through CADD version 1.3^63^. The genomic position of these SNPs and their nearest genes (if available) are shown in Supplementary Table S7. Among these SNPs, rs13191519 is located at an intron region of a RNA gene *XXbac-BPG248L24.13* (also known as *LOC105375015*). Since *LOC105375015* is reported to be associated to HIV and AIDS progression^64^ and HIV is associated to psoriasis, rs13191519 may be associated to psoriasis as well. Meanwhile, rs3094205 is located at the upstream region of *CDSN* and *CDSN* is reported to be associated to psoriasis in an existing literature^65,66^. Therefore these two SNPs are likely to be a associated to psoriasis and may be a new discovery.

### Predicting gene-gene interactions with CADD SNP annotation

By making an assumption that if *SNP*_*i*_ and *SNP*_*j*_ have an SNP-SNP interaction, *Gene*_*i*_ and *Gene*_*j*_ will have a gene-gene interaction where *Gene*_*i*_ and *Gene*_*j*_ are the closest genes to *SNP*_*i*_ and *SNP*_*j*_ respectively, we can predict gene-gene interactions based on the SNP-SNP interactions we have found after annotated with CADD.

### Analysis on common gene-gene interactions predicted by statistically significant SNP-SNP interactions with top 10000th ranking in improvement metric value

We have selected top 10000 statistically significant SNP-SNP interactions in terms of improvement metric value from both datasets. Then we have predicted a number of gene-gene interactions based on these SNP-SNP interactions. As shown in Supplementary Fig. S4, we have predicted 3501 unique gene-gene interactions from dataset phs000019.v1.p1 and 430 unique gene-gene interactions from dataset phs000982.v1.p1. There are 62 common gene-gene interactions between these two datasets. After excluding 2 self-looping interactions found on gene *XXbac-BPG248L24.13* and *CCHCR1*, there are 60 interactions left and are listed in Supplementary Table S8.

**Figure 4:**
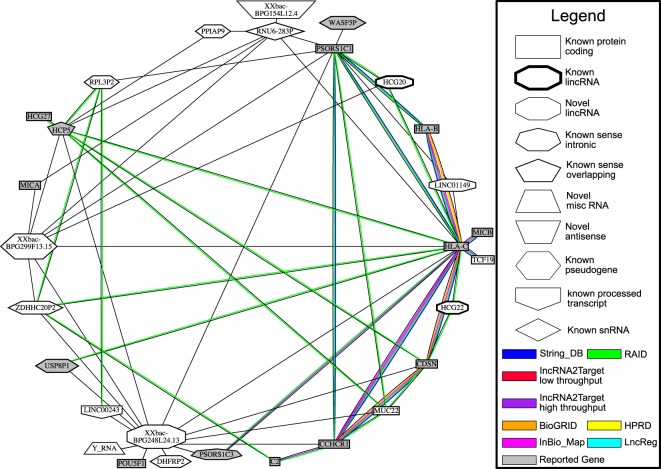
This figure shows a gene network constructed from the 60 common gene-gene interactions predicted from the top 10000 SNP-SNP interactions in terms of improvement metric value found from datasets phs000019.v1.p1 and phs000982.v1.p1 with a circular layout. Genes which are already reported to be associated to psoriasis in existing literatures are coloured in grey colour. Meanwhile, if a gene-gene interaction is supported by a database, a thickened edge with a specific colour will be added to the network.

Among these 60 common gene-gene interactions, there are 29 unique genes. Through referencing Ensembl release 75, the positions of these 29 genes under GRCh37 can be found (see Supplementary Table S9). Among these 29 genes, there are 14 genes which are reported by existing literature to be associated to psoriasis: *HLA-B*^37^, *HLA-C*^37,38^, *PSORS1C1*(which was previous named as SEEK1)^41,42^, *CCHCR1*^67,68^, *HCP5*^39,40^, *CDSN*^65,66^, *USP8P1*^44^, *MICA*^54,69^, *PSORS1C3*^65,70^, *HCG27*^71^, *POU5F1*^72^, *WASF5P*^44^, *MICB*^43^, *C2*^73^. On the other hand, these 60 common gene-gene interactions can be arranged into a network with a circular layout as shown in Fig. 4. Each gene is represented by a node and each common gene-gene interaction is represented by a black edge. If a gene is reported in an existing literature, its corresponding node will be highlighted in grey colour. If a predicted gene-gene interaction is reported as a direct interaction or an indirect interaction through an intermediate gene in one of the following seven biomolecule interaction databases: String^74^, RAID^75^, lncRNA2target^76^, LncReg^77^, InBio_Map^78^, HPRD^79^ and BioGRID^80^, a new edge with a colour corresponding to the reporting database will be added to the network. Furthermore, the gene network can also be arranged with a linear layout (see Supplementary Fig. S5), where the genes are laid down according to their genomic position on Chromosome 6. The recombination rate and SNP pairs with significant linkage disequilibrium score (*r* ≥ 0.9) along Chromosome 6 from position 30734602 to 32233615 are shown in Supplementary Fig. S5a,b respectively. The linkage disequilibrium score is calculated with the samples from phs000982.p1.v1 using Plink^30^.

As seen in Fig. 4, 28 out of 60 common gene-gene interactions predicted can be verified by one or more than one existing databases as direct or indirect gene-gene interactions. Since almost half of the gene-gene interactions are supported by existing databases, our gene network is generally supported. Furthermore, there is a long non-coding RNA (lincRNA) gene *XXbac-BPG248L24.13* acting as a hub and is interacting with eight literature reported psoriasis-associated genes namely *PSORS1C3*, *CDSN*, *POU5F1*, *PSORS1C1*, *CCHCR1*, *HLA-C*, *USP8P1* and *HCP5*. Therefore, *XXbac-BPG248L24.13* may be a new discovery. However, since *XXbac-BPG248L24.13* and two other literature reported psoriasis-associated genes *HLA-C* and *USP8P1* are located in the same LD region as shown in Supplementary Fig. S5b. Therefore, *XXbac-BPG248L24.13* may be a false discovery and the SNPs being mapped to *XXbac-BPG248L24.13* by CADD may be proxy SNPs of the disease-associated SNPs located in *HLA-C* or *USP8P1*. Further verification through wet-lab experiments should be performed. Meanwhile, there is a pseudogene *XXbac-BPG299F13.15* which is located in the same LD region as *HLA-C* and is interacting with 4 other literature reported psoriasis-associated genes (*PSORS1C1*, *MICA*, *HCP5*, *HLA-C*). Similar to *XXbac-BPG248L24.13*, *XXbac-BPG299F13.15* may be a discovery and its effect can only be verified through wet-lab experiments. In Supplementary Fig. S5b, there is long-distance gene-gene interactions between *HCP5* and *MUC22*. Since there is a long-distance haplotype between *HCP5* and *MUC22*, the gene-gene interaction predicted has a high potential to be representing the effect of a long-distance haplotype. Similarly, *LINC00243* and *PSORS1C1* have a predicted gene-gene interaction which corresponds to a long-distance haplotype. Clearly, long-distance disease-associated haplotype can be found through our exhaustive search algorithm as well. Finally, the gene-gene interaction predicted between *XXbac-BPG154L12.4* and *RNU6-283P* may be a novel discovery. Although RNU6-283P and a known psoriasis-associated gene *HLA-B* are both located at the same LD region, *XXbac-BPG154L12.4* is not in any LD region which contains any known psoriasis associated gene. Therefore, *XXbac-BPG154L12.4* may be interacting with a proxy gene of a known psoriasis-associated gene *HLA-B*. Furthermore, there is a strong recombination site in between *XXbac-BPG154L12.4* and any other known psoriasis associated gene. Therefore, the interaction between *XXbac-BPG154L12.4* and *RNU6-283P* cannot be simply explained as a long-distance haplotype and it may be a new discovery.

### Simulations on speeding up counting of contingency table with a pre-computed look-up table

We have performed simulations to compare the time of counting contingency table of each SNP-SNP interaction under every pattern with pre-computed look-up table instead of naively counting the number of cases and controls under black and white genotype under every pattern. In this simulation, we have executed our program under the synthesis datasets generated by us. The average run-time of our program with or without pre-computed look-up table under datasets with different numbers of SNPs and heritabilities are shown in Supplementary Table S10. By observing Supplementary Table S10, we can observe that our program executed with the pre-computed look-up table is at least 8 times faster under datasets with 1000 SNPs and at least 10 times faster under datasets with 5000 and 10000 SNPs. This shows that the pre-computed look-up table is a effective mean to accelerate the counting of contingency table. Since the loading time of the pre-computed look-up table is constant under any dataset inputted, the impact of loading pre-computed look-up table on run-time is far more significant under a smaller dataset. Therefore, the speed-up under datasets with 1000 SNPs is less significant than the speed-up under dataset with 1000 and 10000 SNPs.

## Discussion

This project studies the genetic risk factors of psoriasis. Psoriasis is a chronic inflammatory dermatitis characterised by hyperproliferation of the epidermis. It is a common dermatitis affecting up to 3% of the general population^1^. A strong role of genetic predisposition in its etiology has been confirmed by recent family-based linkage studies and twin studies. Recent GWAS also confirmed a number of predisposition SNPs, particularly in Chromosome 6 *HLA* loci region^1,2^. In this paper, we aim at demonstrating SNP-SNP interactions also play an important role in causing psoriasis. Our results have shown that interactions between SNPs are present in psoriasis patients. Although these interacting SNPs are mostly found in the extended region of HLA in Chromosome 6, there are SNP-SNP interactions spanned across recombination hotspots which excluded the possibility that the epistasis is due to long-distance haplotype effect. Existing gene/protein interaction database also confirmed that the bio-molecule products of these genes in the extended region of *HLA* in Chromosome 6 are indeed interacting with each other.

Performing GWAS through analysing the independent effect of every SNP did not provide an adequate explanation for the hereditability of psoriasis. Previous studies have shown that monozygotic twins were more likely to be affected together than dizygotic twins^81^. Its heritability is as high as 90% which is one of the highest among common diseases and it can equally affect both male and female^82^. On the other hand, the best predisposition SNPs found in *HLA-C* could only increase disease risk by around 4 folds^4,39,83^. This level of odds ratio is not sufficient to account for the high heritability of psoriasis. Other mechanisms must be involved. Gene-gene interactions or gene-environmental interactions are among the most likely explanations for this phenomenon of missing heritability.

**Figure 5:**
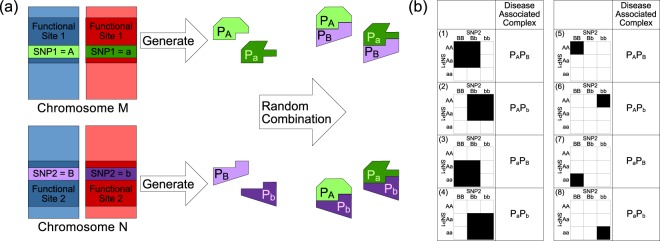
In this figure, part (**a**) shows the bio-molecule interaction mechanism behind a 2^*nd*^ order SNP-SNP interaction^14^ where *SNP*_1_ and *SNP*_2_ are both having genotype (*major*, *minor*). Meanwhile, part (**b**) shows the eight biologically plausible 2^*nd*^ order genotype interaction patterns and their corresponding disease-associated complexes. Major alleles are represented by upper-case letters (i.e. A, B) and minor alleles are represented by lower-case letters (i.e. **a**,**b**).

Unlike other existing GWAS researches, we mainly focus on searching SNP-SNP interactions which can be explained as interactions between two bio-molecules. Although there are many potential mechanisms which can explain the interaction between two loci, interaction between 2 bio-molecules is the most valid and feasible hypothesis. With this idea in mind, we can introduce new constraints to the genotype interaction patterns to shrink the search space. Given any 3 × 3 genotype table of any 2 SNPs, there are total 2^9^ possible genotype interaction patterns available. Such exhaustive enumeration of interaction patterns is unnecessary as most of these patterns are not biologically interpretable under our assumption. Based on our previous analysis on the distribution of bio-molecule complexes between two different bio-molecules, we have shown that the search space can be restricted to only 8 genotype interaction patterns as shown in Fig. 5b. After we have applied our algorithm to two psoriasis GWAS datasets, we have identified interaction between bio-molecules generated from the extended *HLA* region in Chromosome 6.

Among the gene-gene interactions we have found, some are obviously associated to psoriasis. For example, *HLA-C* and *HLA-B*, these 2 genes are probably expressed together and their protein products can interact with antigens on cell surface. It is possible some alleles of the HLA lead to a more intense inflammatory response. For example, *HLA-b27* has been known to be a genetic factor of psoriasis. Similar *HLA* alleles have already been found in other diseases like HIV infection and Diabetes.

Knowledge of interaction between other bio-molecules in the extended *HLA* loci is still less understood. However, genome-wide protein-protein interactions were evident in numerous wet experiment. In fact, it is not uncommon to have interacting gene partners (like ligands and receptors) located in nearby genomic region, with the benefit that they could be regulated simultaneous during organism development. This genomic arrangement leads to various example of gene clusters, like those of cytokines, chemokines and their receptor etc.

## Methods

### Restricting search space of SNP-SNP interaction with biologically plausible genotype interaction pattern

The number of cases and controls under each genotype of any 2^*nd*^ order SNP combination (*SNP*_*i*_, *SNP*_*j*_) can be arranged into a 3 × 3 genotype table similar to the table shown in Fig. 3, where each cell in the 3 × 3 table corresponds to a genotype of (*SNP*_*i*_, *SNP*_*j*_). A genotype in a 3 × 3 table can be labelled as a high-risk or low-risk genotype through applying statistical or heuristic algorithms like multi-factor dimensionality reduction (MDR) algorithm and its derivatives^84^. However, the patterns of the genotype label generated by these algorithms may not be biologically interpretable. In this paper, we have applied eight 2^*nd*^ order biological plausible SNP-SNP interaction patterns^14^ for labelling genotypes into high-risk and low risk genotypes. The principles and assumptions in deriving these eight SNP-SNP interaction patterns are shown in Fig. 5a and are explained below.

1. *SNP*_1_ and *SNP*_2_ are located within two different functional sites *Site*_1_ and *Site*_2_ respectively. Major alleles are represented by upper-case letters (i.e. A, B) and minor alleles are represented by lower-case letters (i.e. a, b).
2. *SNP*_1_ and *SNP*_2_ can affect their respective functional sites and cause each site to produce at most two different subtypes of bio-molecules. For example, bio-molecules *p*_*A*_ is generated from *Site*_1_ with *SNP*_1_ having an major allele.
3. The bio-molecules generated from *Site*_1_ and *Site*_2_ can randomly dock with each other to form at most four different bio-molecule complexes For example, complex *p*_*A*_*p*_*B*_ is composed by bio-molecules *p*_*A*_ and *p*_*B*_ generated from *Site*_1_ and *Site*_2_ respectively.
4. A bio-molecule complex is associated to a genetic disease if (1) Only its solo presence (i.e. no other bio-molecules are present) or (2) Its presence can either promote or inhibit a disease.

The eight biologically plausible SNP-SNP interaction patterns are shown in Fig. 5b. Considering pattern 1 in Fig. 5b, if *p*_*A*_*p*_*B*_ is the only disease-associated bio-molecule complex and its presence can either promote or inhibit a disease (i.e. condition 4b), samples carrying genotype {“*AA*”, “*BB*”}, {“*AA*”, “*Bb*”}, {“*Aa*”, “*BB*”} and {“*Aa*”, “*Bb*”} obviously would have a different level of disease risk comparing to samples carrying other genotypes. After labelling these two groups of genotypes with two different colours, pattern 1 can hence be defined. On the other hand considering pattern 5 in Fig. 5b, if *p*_*A*_*p*_*B*_ is the only disease-associated bio-molecule complex and only its solo presence can either promote or inhibit a disease (i.e. condition 4a), samples carrying genotype AA, BB would have a different level of disease risk comparing to samples with other genotypes. Similarly, other patterns shown in Fig. 5b can be also defined through a similar argument shown above.

Since other genotype interaction patterns are not biologically plausible, we can reduce our search space on genotype interaction patterns from 2^9^ to the eight patterns shown in Fig. 5b and thus significantly reduce the size of the search space.

### Finding statistically significant SNP-SNP interactions with exhaustive search

In Supplementary Fig. S6, the process of converting the 3 × 3 table of a 2^*nd*^ order SNP combination (*SNP*_*i*_, *SNP*_*j*_) into a 2 × 2 contingency table is being demonstrated. Considering a 2^*nd*^ order SNP combination (*SNP*_*i*_, *SNP*_*j*_) after its genotypes being labelled according to genotype interaction pattern 1 in Fig. 5b, the number of cases and controls having black genotypes are summed up as *N*_*D*,*B*_ and *N*_*H*,*B*_ respectively. Meanwhile the number of cases and controls of white genotypes are summed up as *N*_*D*,*W*_ and *N*_*H*,*W*_ respectively. This summation process is shown in Supplementary Fig. S6a. After that, the counts of cases and controls *N*_*D*,*B*_, *N*_*H*,*B*_ and *N*_*D*,*W*_ and *N*_*H*,*W*_ can then be arranged into a 2 × 2 contingency table as shown in Supplementary Fig. S6b. Finally, the 2 × 2 contingency table of every SNP-SNP interaction is subjected to statistical tests and the SNP-SNP interaction found to be statistically significant are analysed. Imputation of SNPs on selected chromosomes were carried out on Michigan Imputation Server (https://imputationserver.sph.umich.edu) which is based on Minimac3 imputation algorithm^85^.

### Accelerating counting of contingency table with a pre-computed look-up table

We propose to accelerate the exhaustive search process through a pre-computed look-up table. For each *SNP*_*i*_, it corresponds to a vector $\overrightarrow{SN{P}_{i}}$ (i.e. the *ith* column of matrix *A* in Table 1). Each vector $\overrightarrow{SN{P}_{i}}$ can be spliced into two different vectors $\overrightarrow{SN{P}_{i,case}}$ and $\overrightarrow{SN{P}_{i,ctrl}}$, where case vector $\overrightarrow{SN{P}_{i,case}}$ only has genotypes of *SNP*_*i*_ from cases and control vector $\overrightarrow{SN{P}_{i,ctrl}}$ has genotypes of $SN{P}_{i}$ from controls. Each genotype can be considered as a 2 bit integer and thus every *p* genotypes in a vector can be combined into a 2*p* bit integer *g*. Given any possible pair of integer *g*, the distribution of black and white genotypes in cases and controls under every genotype interaction pattern can be pre-computed and stored in a look-up table located at the main memory. Therefore, the 2 × 2 contingency table of any pairs of SNP can be found without direct counting. Instead, the distribution of black and white genotypes in cases under any pair of SNP *SNP*_*i*_ and *SNP*_*j*_ can be found by retrieving and summing the distribution of black and white genotypes of every corresponding pair of integer *g* between case vectors $\overrightarrow{SN{P}_{i,case}}$ and $\overrightarrow{SN{P}_{j,case}}$ from the look-up table in the main memory. Meanwhile, the distribution of black and white genotypes in controls can be obtained in a similar fashion. This significantly accelerates the time needed to build the contingency table of each SNP-SNP interaction.

## Electronic supplementary material

Supplementary Material
