# Supplementary material for: Discovering Genetic Factors for psoriasis through exhaustively searching for significant second order SNP-SNP interactions
Source: Sci Rep. 2018 Oct 12;8:15186. doi: 10.1038/s41598-018-33493-w (PMC6185942; doi:10.1038/s41598-018-33493-w)
Supplement: Supplementary file 1 — Supplementary Material [file 41598_2018_33493_MOESM1_ESM.pdf]

Supplementary Materials

Title: Discovering Genetic Factors for psoriasis through exhaustively searching for significant second order SNP-SNP interactions

Authors: Kwan-Yeung Lee, Kwong-Sak Leung, Nelson L.S. Tang and Man-Hon Wong

| | phs000019.v1.p1 | phs000982.v1.p1 |
|---|---|---|
| Missing rate | >0.01 | >0.05 |
| Minor allele frequency (MAF) | <0.01 | |
| Hardy–Weinberg equilibrium (HWE) | $p$-value<0.00001 | |
| Missing rate deviation between cases and controls | $p$-value<0.00001 | |

**Supplementary Table S1:** This table shows the data cleansing parameters for filtering low-quality SNPs. SNPs satisfying any one of the following conditions listed in this table are filtered.

| | phs000019.v1.p1 | phs000982.v1.p1 |
|---|---|---|
| Missing rate | >0.05 | >0.005 |
| Gender | Inconsistency between reported gender and gender predicted with X chromosome | |
| Heterozygosity rate | - | <0.2775 or >0.2875 |

**Supplementary Table S2:** This table shows the data cleansing parameters for filtering low-quality samples. Samples satisfying any one of the following conditions listed in this table are filtered.

| SNP1 | SNP2 | Pattern | SNP1 $p$-value | SNP2 $p$-value | $2^{nd}$ Order $p$-value | Improvement Metric Value | Odds Ratio | $2^{nd}$ Order $p$-value (phs000982) |
|---|---|---|---|---|---|---|---|---|
| rs12191877 | rs2534666 | 4 | $6.89 \times 10^{-23}$ | $2.08 \times 10^{-6}$ | $6.93 \times 10^{-30}$ | $9.95 \times 10^{6}$ | 3.9339 | $1.28 \times 10^{-14}$ |
| rs3130573 | rs12191877 | 4 | $6.76 \times 10^{-8}$ | $6.89 \times 10^{-23}$ | $6.98 \times 10^{-29}$ | $9.87 \times 10^{5}$ | 3.7570 | $8.04 \times 10^{-17}$ |
| rs9262492 | rs12191877 | 4 | $4.39 \times 10^{-6}$ | $6.89 \times 10^{-23}$ | $3.28 \times 10^{-28}$ | $2.10 \times 10^{5}$ | 3.5653 | $2.75 \times 10^{-26}$ |
| rs2894176 | rs12191877 | 4 | $1.93 \times 10^{-6}$ | $6.89 \times 10^{-23}$ | $3.64 \times 10^{-28}$ | $1.89 \times 10^{5}$ | 3.5434 | $1.40 \times 10^{-25}$ |
| rs3130517 | rs12191877 | 4 | $3.57 \times 10^{-17}$ | $6.89 \times 10^{-23}$ | $4.45 \times 10^{-28}$ | $1.55 \times 10^{5}$ | 3.5369 | $1.51 \times 10^{-18}$ |
| rs9262498 | rs12191877 | 4 | $3.90 \times 10^{-6}$ | $6.89 \times 10^{-23}$ | $5.01 \times 10^{-28}$ | $1.38 \times 10^{5}$ | 3.5473 | $3.65 \times 10^{-26}$ |
| rs1265078 | rs12191877 | 4 | $2.40 \times 10^{-11}$ | $6.89 \times 10^{-23}$ | $6.80 \times 10^{-28}$ | $1.01 \times 10^{5}$ | 3.5547 | $1.81 \times 10^{-18}$ |
| rs12191877 | rs2516417 | 3 | $6.89 \times 10^{-23}$ | $6.72 \times 10^{-4}$ | $8.30 \times 10^{-28}$ | $8.31 \times 10^{4}$ | 3.3355 | $1.34 \times 10^{-20}$ |
| rs12191877 | rs2844502 | 3 | $6.89 \times 10^{-23}$ | $9.32 \times 10^{-3}$ | $1.35 \times 10^{-27}$ | $5.12 \times 10^{4}$ | 3.3261 | $8.76 \times 10^{-20}$ |
| rs3130467 | rs12191877 | 4 | $2.81 \times 10^{-16}$ | $6.89 \times 10^{-23}$ | $1.57 \times 10^{-27}$ | $4.40 \times 10^{4}$ | 3.4868 | $1.66 \times 10^{-18}$ |
| rs12191877 | rs2516510 | 3 | $6.89 \times 10^{-23}$ | $1.04 \times 10^{-2}$ | $1.90 \times 10^{-27}$ | $3.62 \times 10^{4}$ | 3.3109 | $1.54 \times 10^{-20}$ |
| rs3130517 | rs2894207 | 4 | $3.57 \times 10^{-17}$ | $1.28 \times 10^{-15}$ | $2.14 \times 10^{-27}$ | $1.67 \times 10^{10}$ | 3.4250 | $5.65 \times 10^{-18}$ |
| rs3094205 | rs12191877 | 4 | $2.29 \times 10^{-9}$ | $6.89 \times 10^{-23}$ | $3.97 \times 10^{-27}$ | $1.74 \times 10^{4}$ | 3.4440 | $6.78 \times 10^{-16}$ |
| rs2244027 | rs12191877 | 2 | $4.46 \times 10^{-2}$ | $6.89 \times 10^{-23}$ | $4.28 \times 10^{-27}$ | $1.61 \times 10^{4}$ | 3.2691 | $3.23 \times 10^{-20}$ |
| rs12191877 | rs10848821 | 3 | $6.89 \times 10^{-23}$ | $2.27 \times 10^{-2}$ | $6.76 \times 10^{-27}$ | $1.02 \times 10^{4}$ | 3.3101 | $3.26 \times 10^{-20}$ |
| rs3130467 | rs2894207 | 4 | $2.81 \times 10^{-16}$ | $1.28 \times 10^{-15}$ | $7.28 \times 10^{-27}$ | $3.86 \times 10^{10}$ | 3.3781 | $6.15 \times 10^{-18}$ |
| rs12191877 | rs2523708 | 3 | $6.89 \times 10^{-23}$ | $1.60 \times 10^{-2}$ | $8.76 \times 10^{-27}$ | $7.87 \times 10^{3}$ | 3.2564 | $2.13 \times 10^{-20}$ |
| rs12191877 | rs10483336 | 3 | $6.89 \times 10^{-23}$ | $2.40 \times 10^{-4}$ | $9.21 \times 10^{-27}$ | $7.49 \times 10^{3}$ | 3.2045 | $9.55 \times 10^{-23}$ |
| rs12191877 | rs6075938 | 3 | $6.89 \times 10^{-23}$ | $7.79 \times 10^{-3}$ | $1.15 \times 10^{-26}$ | $5.98 \times 10^{3}$ | 3.2515 | $1.58 \times 10^{-20}$ |
| rs1265078 | rs2894207 | 4 | $2.40 \times 10^{-11}$ | $1.28 \times 10^{-15}$ | $1.43 \times 10^{-26}$ | $8.94 \times 10^{10}$ | 3.3389 | $6.79 \times 10^{-18}$ |

**Supplementary Table S3:** This table shows the top 20 statistically significant $2^{nd}$ order SNP-SNP interactions in terms of $p$-value from psoriasis dataset phs000019.v1.p1.

| SNP1 | SNP2 | Pattern | SNP1 $p$-value | SNP2 $p$-value | $2^{nd}$ Order $p$-value | Improvement Metric Value | Odds Ratio | $2^{nd}$ Order $p$-value (phs000019) |
|---|---|---|---|---|---|---|---|---|
| rs13203895 | rs4349859 | 5 | $6.79 \times 10^{-26}$ | $7.20 \times 10^{-9}$ | $1.18 \times 10^{-34}$ | $5.77 \times 10^{8}$ | 2.6638 | $3.14 \times 10^{-21}$ |
| rs13191519 | rs4349859 | 5 | $4.07 \times 10^{-26}$ | $7.20 \times 10^{-9}$ | $1.26 \times 10^{-34}$ | $3.23 \times 10^{8}$ | 2.6829 | $8.25 \times 10^{-21}$ |
| rs13203895 | rs4418214 | 5 | $6.79 \times 10^{-26}$ | $2.02 \times 10^{-19}$ | $1.67 \times 10^{-34}$ | $4.06 \times 10^{8}$ | 2.6480 | $1.79 \times 10^{-19}$ |
| rs13191519 | rs4418214 | 5 | $4.07 \times 10^{-26}$ | $2.02 \times 10^{-19}$ | $1.86 \times 10^{-34}$ | $2.18 \times 10^{8}$ | 2.6651 | $6.38 \times 10^{-19}$ |
| rs10484554 | rs4349859 | 5 | $9.96 \times 10^{-26}$ | $7.20 \times 10^{-9}$ | $4.17 \times 10^{-34}$ | $2.39 \times 10^{8}$ | 2.6451 | $7.11 \times 10^{-21}$ |
| rs10484554 | rs4418214 | 5 | $9.96 \times 10^{-26}$ | $2.02 \times 10^{-19}$ | $5.89 \times 10^{-34}$ | $1.69 \times 10^{8}$ | 2.6294 | $5.56 \times 10^{-19}$ |
| rs13203895 | rs45533135 | 5 | $6.79 \times 10^{-26}$ | $1.20 \times 10^{-8}$ | $7.72 \times 10^{-34}$ | $8.80 \times 10^{7}$ | 2.6206 | $8.50 \times 10^{-20}$ |
| rs13191519 | rs45533135 | 5 | $4.07 \times 10^{-26}$ | $1.20 \times 10^{-8}$ | $8.72 \times 10^{-34}$ | $4.67 \times 10^{7}$ | 2.6371 | $1.76 \times 10^{-19}$ |
| rs75851973 | rs13203895 | 5 | $8.19 \times 10^{-10}$ | $6.79 \times 10^{-26}$ | $2.05 \times 10^{-33}$ | $3.31 \times 10^{7}$ | 2.6087 | $1.13 \times 10^{-21}$ |
| rs76956521 | rs13203895 | 5 | $8.19 \times 10^{-10}$ | $6.79 \times 10^{-26}$ | $2.05 \times 10^{-33}$ | $3.31 \times 10^{7}$ | 2.6087 | $1.13 \times 10^{-21}$ |
| rs17728338 | rs13203895 | 5 | $1.59 \times 10^{-9}$ | $6.79 \times 10^{-26}$ | $2.14 \times 10^{-33}$ | $3.17 \times 10^{7}$ | 2.6073 | $6.55 \times 10^{-22}$ |
| rs75851973 | rs13191519 | 5 | $8.19 \times 10^{-10}$ | $4.07 \times 10^{-26}$ | $2.26 \times 10^{-33}$ | $1.80 \times 10^{7}$ | 2.6260 | $1.85 \times 10^{-20}$ |
| rs76956521 | rs13191519 | 5 | $8.19 \times 10^{-10}$ | $4.07 \times 10^{-26}$ | $2.26 \times 10^{-33}$ | $1.80 \times 10^{7}$ | 2.6260 | $1.85 \times 10^{-20}$ |
| rs17728338 | rs13191519 | 5 | $1.59 \times 10^{-9}$ | $4.07 \times 10^{-26}$ | $2.37 \times 10^{-33}$ | $1.72 \times 10^{7}$ | 2.6244 | $1.07 \times 10^{-20}$ |
| snp5-150450166 | rs13203895 | 5 | $1.25 \times 10^{-9}$ | $6.79 \times 10^{-26}$ | $2.47 \times 10^{-33}$ | $2.75 \times 10^{7}$ | 2.6058 | — |
| rs10484554 | rs45533135 | 5 | $9.96 \times 10^{-26}$ | $1.20 \times 10^{-8}$ | $2.69 \times 10^{-33}$ | $3.70 \times 10^{7}$ | 2.6021 | $1.53 \times 10^{-19}$ |
| snp5-150450166 | rs13191519 | 5 | $1.25 \times 10^{-9}$ | $4.07 \times 10^{-26}$ | $2.72 \times 10^{-33}$ | $1.50 \times 10^{7}$ | 2.6231 | — |
| rs75851973 | rs10484554 | 5 | $8.19 \times 10^{-10}$ | $9.96 \times 10^{-26}$ | $4.74 \times 10^{-33}$ | $2.10 \times 10^{7}$ | 2.5966 | $9.32 \times 10^{-21}$ |
| rs76956521 | rs10484554 | 5 | $8.19 \times 10^{-10}$ | $9.96 \times 10^{-26}$ | $4.74 \times 10^{-33}$ | $2.10 \times 10^{7}$ | 2.5966 | $9.32 \times 10^{-21}$ |
| rs17728338 | rs10484554 | 5 | $1.59 \times 10^{-9}$ | $9.96 \times 10^{-26}$ | $4.95 \times 10^{-33}$ | $2.01 \times 10^{7}$ | 2.5952 | $5.40 \times 10^{-21}$ |

**Supplementary Table S4:** This table shows the top 20 statistically significant $2^{nd}$ order SNP-SNP interactions in terms of $p$-value from psoriasis dataset phs000982.v1.p1.

| SNP1 | SNP2 | Pattern | SNP1 $p$-value | SNP2 $p$-value | $2^{nd}$ Order $p$-value | Improvement Metric Value | Odds Ratio | $2^{nd}$ Order $p$-value (phs000982) |
|---|---|---|---|---|---|---|---|---|
| rs1265078 | rs7773175 | 4 | $2.40 \times 10^{-11}$ | $3.04 \times 10^{-13}$ | $4.69 \times 10^{-25}$ | $6.48 \times 10^{11}$ | 3.0436 | $6.50 \times 10^{-16}$ |
| rs1265078 | rs2894207 | 4 | $2.40 \times 10^{-11}$ | $1.28 \times 10^{-15}$ | $1.43 \times 10^{-26}$ | $8.94 \times 10^{10}$ | 3.3389 | $6.79 \times 10^{-18}$ |
| rs3130573 | rs7773175 | 4 | $6.76 \times 10^{-8}$ | $3.04 \times 10^{-13}$ | $5.68 \times 10^{-24}$ | $5.35 \times 10^{10}$ | 3.0176 | $1.79 \times 10^{-13}$ |
| rs9380237 | rs2534666 | 4 | $5.59 \times 10^{-10}$ | $2.08 \times 10^{-6}$ | $1.11 \times 10^{-20}$ | $5.02 \times 10^{10}$ | 2.7506 | $1.12 \times 10^{-9}$ |
| rs3130467 | rs2894207 | 4 | $2.81 \times 10^{-16}$ | $1.28 \times 10^{-15}$ | $7.28 \times 10^{-27}$ | $3.86 \times 10^{10}$ | 3.3781 | $6.15 \times 10^{-18}$ |
| rs7756521 | rs1265078 | 4 | $4.65 \times 10^{-9}$ | $2.40 \times 10^{-11}$ | $7.98 \times 10^{-22}$ | $3.00 \times 10^{10}$ | 3.1234 | $1.22 \times 10^{-11}$ |
| rs1265078 | rs9380237 | 4 | $2.40 \times 10^{-11}$ | $5.59 \times 10^{-10}$ | $9.45 \times 10^{-22}$ | $2.53 \times 10^{10}$ | 2.8208 | $2.75 \times 10^{-18}$ |
| rs3130517 | rs2894207 | 4 | $3.57 \times 10^{-17}$ | $1.28 \times 10^{-15}$ | $2.14 \times 10^{-27}$ | $1.67 \times 10^{10}$ | 3.4250 | $5.65 \times 10^{-18}$ |
| rs2894207 | rs2534666 | 4 | $1.28 \times 10^{-15}$ | $2.08 \times 10^{-6}$ | $1.09 \times 10^{-25}$ | $1.17 \times 10^{10}$ | 3.3436 | $4.22 \times 10^{-12}$ |
| rs3130517 | rs7773175 | 4 | $3.57 \times 10^{-17}$ | $3.04 \times 10^{-13}$ | $1.45 \times 10^{-26}$ | $2.46 \times 10^{9}$ | 3.1788 | $2.10 \times 10^{-15}$ |
| rs2853950 | rs3130573 | 4 | $3.93 \times 10^{-6}$ | $6.76 \times 10^{-8}$ | $2.93 \times 10^{-17}$ | $2.30 \times 10^{9}$ | 2.4168 | $4.15 \times 10^{-8}$ |
| rs3094205 | rs1265078 | 4 | $2.29 \times 10^{-9}$ | $2.40 \times 10^{-11}$ | $1.37 \times 10^{-20}$ | $1.75 \times 10^{9}$ | 2.6799 | $6.27 \times 10^{-13}$ |
| rs7756521 | rs8365 | 4 | $4.65 \times 10^{-9}$ | $1.29 \times 10^{-8}$ | $3.36 \times 10^{-18}$ | $1.38 \times 10^{9}$ | 3.3442 | $3.03 \times 10^{-6}$ |
| rs3130467 | rs7773175 | 4 | $2.81 \times 10^{-16}$ | $3.04 \times 10^{-13}$ | $2.08 \times 10^{-25}$ | $1.35 \times 10^{9}$ | 3.0823 | $3.11 \times 10^{-15}$ |
| rs2894176 | rs3130467 | 4 | $1.93 \times 10^{-6}$ | $2.81 \times 10^{-16}$ | $2.61 \times 10^{-25}$ | $1.08 \times 10^{9}$ | 3.0994 | $4.16 \times 10^{-13}$ |
| rs7756521 | rs3130573 | 4 | $4.65 \times 10^{-9}$ | $6.76 \times 10^{-8}$ | $5.13 \times 10^{-18}$ | $9.07 \times 10^{8}$ | 2.7101 | $1.04 \times 10^{-8}$ |
| rs2894176 | rs3130517 | 4 | $1.93 \times 10^{-6}$ | $3.57 \times 10^{-17}$ | $5.38 \times 10^{-26}$ | $6.64 \times 10^{8}$ | 3.1598 | $2.12 \times 10^{-13}$ |
| rs9262498 | rs1265078 | 4 | $3.90 \times 10^{-6}$ | $2.40 \times 10^{-11}$ | $3.71 \times 10^{-20}$ | $6.46 \times 10^{8}$ | 2.6695 | $4.27 \times 10^{-11}$ |
| rs2844645 | rs3094205 | 2 | $2.56 \times 10^{-9}$ | $2.29 \times 10^{-9}$ | $3.72 \times 10^{-18}$ | $6.14 \times 10^{8}$ | 2.4554 | $1.42 \times 10^{-7}$ |
| rs3130573 | rs2894207 | 4 | $6.76 \times 10^{-8}$ | $1.28 \times 10^{-15}$ | $2.26 \times 10^{-24}$ | $5.66 \times 10^{8}$ | 3.1827 | $1.81 \times 10^{-15}$ |

**Supplementary Table S5:** This table shows the top 20 statistically significant $2^{nd}$ order SNP-SNP interactions in terms of improvement metric value from psoriasis dataset phs000019.v1.p1.

| SNP1 | SNP2 | Pattern | SNP1 $p$-value | SNP2 $p$-value | $2^{nd}$ Order $p$-value | Improvement Metric Value | Odds Ratio | $2^{nd}$ Order $p$-value (phs000019) |
|---|---|---|---|---|---|---|---|---|
| rs2517985 | rs4358666 | 5 | $5.02 \times 10^{-8}$ | $2.62 \times 10^{-9}$ | $1.07 \times 10^{-21}$ | $2.45 \times 10^{12}$ | 2.3225 | $6.84 \times 10^{-11}$ |
| rs2517985 | rs34100370 | 5 | $5.02 \times 10^{-8}$ | $2.62 \times 10^{-9}$ | $1.07 \times 10^{-21}$ | $2.45 \times 10^{12}$ | 2.3225 | $4.55 \times 10^{-11}$ |
| rs2517985 | rs2395491 | 5 | $5.02 \times 10^{-8}$ | $5.01 \times 10^{-8}$ | $3.46 \times 10^{-20}$ | $1.45 \times 10^{12}$ | 2.2707 | $3.04 \times 10^{-9}$ |
| rs2517985 | rs4624908 | 5 | $5.02 \times 10^{-8}$ | $7.03 \times 10^{-8}$ | $3.83 \times 10^{-20}$ | $1.31 \times 10^{12}$ | 2.268 69 | $3.04 \times 10^{-9}$ |
| rs2517985 | rs7754026 | 5 | $5.02 \times 10^{-8}$ | $6.74 \times 10^{-8}$ | $4.41 \times 10^{-20}$ | $1.14 \times 10^{12}$ | 2.2664 | $3.04 \times 10^{-9}$ |
| rs2517985 | rs13194571 | 5 | $5.02 \times 10^{-8}$ | $4.98 \times 10^{-8}$ | $4.95 \times 10^{-20}$ | $1.00 \times 10^{12}$ | 2.2637 | $3.04 \times 10^{-9}$ |
| rs2517985 | rs7775117 | 5 | $5.02 \times 10^{-8}$ | $6.27 \times 10^{-8}$ | $5.03 \times 10^{-20}$ | $9.99 \times 10^{11}$ | 2.2632 | $3.04 \times 10^{-9}$ |
| rs1265079 | rs4358666 | 5 | $7.09 \times 10^{-8}$ | $2.62 \times 10^{-9}$ | $3.16 \times 10^{-21}$ | $8.30 \times 10^{11}$ | 2.3042 | $4.77 \times 10^{-11}$ |
| rs1265079 | rs34100370 | 5 | $7.09 \times 10^{-8}$ | $2.62 \times 10^{-9}$ | $3.16 \times 10^{-21}$ | $8.30 \times 10^{11}$ | 2.3042 | $3.33 \times 10^{-11}$ |
| rs1265079 | rs4624908 | 5 | $7.09 \times 10^{-8}$ | $7.03 \times 10^{-8}$ | $1.11 \times 10^{-19}$ | $6.33 \times 10^{11}$ | 2.2501 | $2.17 \times 10^{-9}$ |
| rs746647 | rs4358666 | 5 | $9.60 \times 10^{-8}$ | $2.62 \times 10^{-9}$ | $4.62 \times 10^{-21}$ | $5.67 \times 10^{11}$ | 2.2955 | $4.77 \times 10^{-11}$ |
| rs746647 | rs34100370 | 5 | $9.60 \times 10^{-8}$ | $2.62 \times 10^{-9}$ | $4.62 \times 10^{-21}$ | $5.67 \times 10^{11}$ | 2.2955 | $3.33 \times 10^{-11}$ |
| rs1576 | rs4358666 | 5 | $9.60 \times 10^{-8}$ | $2.62 \times 10^{-9}$ | $4.62 \times 10^{-21}$ | $5.67 \times 10^{11}$ | 2.2955 | $7.19 \times 10^{-11}$ |
| rs1576 | rs34100370 | 5 | $9.60 \times 10^{-8}$ | $2.62 \times 10^{-9}$ | $4.62 \times 10^{-21}$ | $5.67 \times 10^{11}$ | 2.2955 | $4.93 \times 10^{-11}$ |
| rs1265114 | rs4358666 | 5 | $9.60 \times 10^{-8}$ | $2.62 \times 10^{-9}$ | $4.62 \times 10^{-21}$ | $5.67 \times 10^{11}$ | 2.2955 | $6.84 \times 10^{-11}$ |
| rs1265114 | rs34100370 | 5 | $9.60 \times 10^{-8}$ | $2.62 \times 10^{-9}$ | $4.62 \times 10^{-21}$ | $5.67 \times 10^{11}$ | 2.2955 | $4.55 \times 10^{-11}$ |
| rs1265079 | rs7754026 | 5 | $7.09 \times 10^{-8}$ | $6.74 \times 10^{-8}$ | $1.28 \times 10^{-19}$ | $5.27 \times 10^{11}$ | 2.2477 | $2.17 \times 10^{-9}$ |
| rs1265112 | rs4358666 | 5 | $8.43 \times 10^{-8}$ | $2.62 \times 10^{-9}$ | $5.14 \times 10^{-21}$ | $5.10 \times 10^{11}$ | 2.2934 | $6.84 \times 10^{-11}$ |
| rs1265112 | rs34100370 | 5 | $8.43 \times 10^{-8}$ | $2.62 \times 10^{-9}$ | $5.14 \times 10^{-21}$ | $5.10 \times 10^{11}$ | 2.2934 | $4.55 \times 10^{-11}$ |
| rs1265067 | rs4358666 | 5 | $1.05 \times 10^{-7}$ | $2.62 \times 10^{-9}$ | $5.14 \times 10^{-21}$ | $5.10 \times 10^{11}$ | 2.2934 | $6.84 \times 10^{-11}$ |

**Supplementary Table S6:** This table shows the top 20 statistically significant $2^{nd}$ order SNP-SNP interactions in terms of improvement metric value from psoriasis dataset phs000982.v1.p1.

| SNP | Chr | Position | Annotation | Consequence | GeneName | GeneType |
|---|---|---|---|---|---|---|
| rs10483336 | 14 | 28837590 | Transcript | INTRONIC | CTD-2591A6.2 | lincRNA |
| rs10484554 | 6 | 31274555 | Intergenic | DOWNSTREAM | XXbac-BPG248L24.10 | pseudogene |
| rs10848821 | 12 | 3298031 | Transcript | INTRONIC | TSPAN9 | protein coding |
| rs12191877 | 6 | 31252925 | Intergenic | DOWNSTREAM | RPL3P2 | pseudogene |
| rs1265067 | 6 | 31116142 | Transcript | INTRONIC | CCHCR1 | protein coding |
| rs1265078 | 6 | 31112602 | Intergenic | DOWNSTREAM | PSORS1C1 | protein coding |
| rs1265079 | 6 | 31112108 | Intergenic | UPSTREAM | PSORS1C2 | protein coding |
| rs1265112 | 6 | 31118019 | Transcript | INTRONIC | CCHCR1 | protein coding |
| rs1265114 | 6 | 31117188 | Transcript | INTRONIC | CCHCR1 | protein coding |
| rs13191519 | 6 | 31265752 | Transcript | INTRONIC | XXbac-BPG248L24.13 | lincRNA |
| rs13194571 | 6 | 31342960 | Intergenic | DOWNSTREAM | RNU6-283P | snRNA |
| rs13203895 | 6 | 31244082 | Non-Coding Transcript | NONCODING_CHANGE | USP8P1 | pseudogene |
| rs1576 | 6 | 31110391 | Regulatory Feature | NON_SYNONYMOUS | CCHCR1 | protein coding |
| rs17728338 | 5 | 150478318 | Intergenic | DOWNSTREAM | ANXA6 | protein coding |
| rs2244027 | 6 | 31347566 | Intergenic | UPSTREAM | ZDHHC20P2 | pseudogene |
| rs2395491 | 6 | 31342420 | Intergenic | DOWNSTREAM | RNU6-283P | snRNA |
| rs2516417 | 6 | 31459636 | Regulatory Feature | REGULATORY | - | - |
| rs2516510 | 6 | 31449914 | Intergenic | DOWNSTREAM | HCP5 | sense overlapping |
| rs2517985 | 6 | 31118942 | Transcript | INTRONIC | CCHCR1 | protein coding |
| rs2523708 | 6 | 31451279 | Intergenic | INTERGENIC | - | - |
| rs2534666 | 6 | 31468546 | Intergenic | UPSTREAM | Y_RNA | misc RNA |
| rs2844502 | 6 | 31445216 | Non-Coding Transcript | NONCODING_CHANGE | HCP5 | sense overlapping |
| rs2844645 | 6 | 31015182 | Intergenic | INTERGENIC | - | - |
| rs2853950 | 6 | 31236175 | Intergenic | DOWNSTREAM | HLA-C | protein coding |
| rs2894176 | 6 | 30986038 | Transcript | INTRONIC | MUC22 | protein coding |
| rs2894207 | 6 | 31263751 | Transcript | INTRONIC | XXbac-BPG248L24.13 | lincRNA |
| rs3094205 | 6 | 31091862 | Intergenic | UPSTREAM | CDSN | protein coding |
| rs3130467 | 6 | 31187075 | Intergenic | UPSTREAM | XXbac-BPG299F13.15 | pseudogene |
| rs3130517 | 6 | 31190303 | Intergenic | UPSTREAM | XXbac-BPG299F13.15 | pseudogene |
| rs3130573 | 6 | 31106268 | Intergenic | DOWNSTREAM | CCHCR1 | protein coding |
| rs34100370 | 6 | 31339937 | Intergenic | DOWNSTREAM | RNU6-283P | snRNA |
| rs4349859 | 6 | 31365787 | Regulatory Feature | REGULATORY | - | - |
| rs4358666 | 6 | 31340477 | Intergenic | DOWNSTREAM | RNU6-283P | snRNA |
| rs4418214 | 6 | 31391401 | Transcript | INTRONIC | HCP5 | sense overlapping |
| rs45533135 | 6 | 31430951 | Non-Coding Transcript | NONCODING_CHANGE | HCP5 | sense overlapping |
| rs4624908 | 6 | 31342827 | Intergenic | DOWNSTREAM | RNU6-283P | snRNA |
| rs6075938 | 20 | 22689686 | Intergenic | INTERGENIC | - | - |
| rs746647 | 6 | 31114182 | Transcript | INTRONIC | CCHCR1 | protein coding |
| rs7754026 | 6 | 31345064 | Intergenic | UPSTREAM | ZDHHC20P2 | pseudogene |
| rs7756521 | 6 | 30848253 | Intergenic | UPSTREAM | DDR1 | protein coding |
| rs7773175 | 6 | 31240959 | Intergenic | UPSTREAM | HLA-C | protein coding |
| rs7775117 | 6 | 31345486 | Coding Transcript | NONCODING_CHANGE | FGFR3P1 | pseudogene |
| rs8365 | 6 | 32148403 | Intergenic | DOWNSTREAM | PBX2 | protein coding |
| rs9262492 | 6 | 30986015 | Transcript | INTRONIC | MUC22 | protein coding |
| rs9262498 | 6 | 30986835 | Transcript | INTRONIC | MUC22 | protein coding |
| rs9380237 | 6 | 31264392 | Transcript | INTRONIC | XXbac-BPG248L24.13 | lincRNA |

**Supplementary Table S7:** This table shows the genomic position and the nearest gene of every SNP in the top 20 SNP-SNP interactions in terms of $p$-value or improvement metric value from both datasets.

| Gene1 | Gene2 | phs000019.v1.p1 | | | | phs000982.v1.p1 | | | |
|---|---|---|---|---|---|---|---|---|---|
| | | SNP1,SNP2,Pattern | Pairwise Pval | Improvement Metric Value | Odds Ratio | SNP1,SNP2,Pattern | Pairwise Pval | Improvement Metric Value | Odds Ratio |
| HLA-C | PSORS1C1 | rs1265078,rs7773175,4 | $4.69 \times 10^{-25}$ | $6.48 \times 10^{11}$ | 3.0436 | rs6911408,rs2853953,4 | $3.51 \times 10^{-28}$ | $1.30 \times 10^{9}$ | 2.5349 |
| PSORS1C1 | XXbac-BPG248L24.13 | rs1265078,rs2894207,4 | $1.43 \times 10^{-26}$ | $8.94 \times 10^{10}$ | 3.3389 | rs6911408,rs9468932,4 | $2.00 \times 10^{-28}$ | $4.77 \times 10^{10}$ | 2.5974 |
| CCHCR1 | HLA-C | rs3130573,rs7773175,4 | $5.68 \times 10^{-24}$ | $5.35 \times 10^{10}$ | 3.0176 | rs3130453,rs2249742,2 | $7.09 \times 10^{-18}$ | $1.11 \times 10^{9}$ | 1.9954 |
| XXbac-BPG248L24.13 | Y_RNA | rs9380237,rs2534666,4 | $1.11 \times 10^{-20}$ | $5.02 \times 10^{10}$ | 2.7506 | rs9380237,rs2596542,4 | $6.23 \times 10^{-20}$ | $6.53 \times 10^{6}$ | 2.1035 |
| XXbac-BPG248L24.13 | XXbac-BPG299F13.15 | rs3130467,rs2894207,4 | $7.28 \times 10^{-27}$ | $3.86 \times 10^{10}$ | 3.3781 | rs1639110,rs9380238,4 | $6.25 \times 10^{-19}$ | $3.34 \times 10^{7}$ | 2.2810 |
| HLA-C | XXbac-BPG299F13.15 | rs3130517,rs7773175,4 | $1.45 \times 10^{-26}$ | $2.46 \times 10^{9}$ | 3.1788 | rs6933647,rs2524096,5 | $5.16 \times 10^{-18}$ | $5.07 \times 10^{6}$ | 2.5830 |
| MUC22 | PSORS1C1 | rs9262498,rs1265078,4 | $3.71 \times 10^{-20}$ | $6.46 \times 10^{8}$ | 2.6695 | rs7776233,rs9263717,4 | $1.99 \times 10^{-22}$ | $6.19 \times 10^{6}$ | 2.2224 |
| CCHCR1 | XXbac-BPG248L24.13 | rs3130573,rs2894207,4 | $2.26 \times 10^{-24}$ | $5.66 \times 10^{8}$ | 3.1827 | rs130076,rs9380238,4 | $4.02 \times 10^{-18}$ | $1.40 \times 10^{7}$ | 2.2358 |
| PSORS1C1 | RNU6-283P | rs2844580,rs1265078,2 | $6.06 \times 10^{-14}$ | 394.984 | 2.1622 | rs1265078,rs4624908,5 | $1.43 \times 10^{-19}$ | $4.90 \times 10^{11}$ | 2.2451 |
| CDSN | XXbac-BPG248L24.13 | rs3094205,rs9380237,4 | $2.96 \times 10^{-18}$ | $1.89 \times 10^{8}$ | 2.5056 | rs3130982,rs9366778,5 | $5.54 \times 10^{-16}$ | $3.63 \times 10^{6}$ | 3.1811 |
| MICA | RNU6-283P | rs9266490,rs6933779,4 | $6.74 \times 10^{-15}$ | $8.79 \times 10^{7}$ | 2.9467 | rs4358666,rs9468992,5 | $4.52 \times 10^{-19}$ | $4.74 \times 10^{6}$ | 2.0073 |
| CCHCR1 | CDSN | rs3094205,rs3130573,4 | $2.89 \times 10^{-17}$ | $7.90 \times 10^{7}$ | 2.4316 | rs6913137,rs130069,5 | $3.62 \times 10^{-22}$ | $1.82 \times 10^{6}$ | 2.2245 |
| CCHCR1 | MUC22 | rs9262498,rs3130573,4 | $9.46 \times 10^{-16}$ | $7.15 \times 10^{7}$ | 2.3177 | rs13210132,rs130069,5 | $1.27 \times 10^{-19}$ | $2.27 \times 10^{6}$ | 2.2636 |
| MUC22 | XXbac-BPG248L24.13 | rs9262492,rs2894207,4 | $2.86 \times 10^{-23}$ | $4.47 \times 10^{7}$ | 3.0012 | rs7776233,rs1960278,4 | $4.34 \times 10^{-15}$ | $6.28 \times 10^{8}$ | 1.8616 |
| HLA-C | XXbac-BPG248L24.13 | rs3132486,rs9380237,2 | $1.89 \times 10^{-17}$ | $2.96 \times 10^{7}$ | 2.4126 | rs2853952,rs9468932,4 | $6.31 \times 10^{-28}$ | $6.98 \times 10^{8}$ | 2.5409 |
| CDSN | MUC22 | rs9394031,rs3094205,4 | $1.01 \times 10^{-16}$ | $5.85 \times 10^{6}$ | 2.9664 | rs7776233,rs2233959,4 | $1.54 \times 10^{-15}$ | $1.33 \times 10^{6}$ | 1.8673 |
| HCG27 | HCP5 | rs7762933,rs9501106,2 | $4.19 \times 10^{-15}$ | $4.85 \times 10^{6}$ | 2.5424 | rs3132506,rs45533135,5 | $5.56 \times 10^{-20}$ | $5.90 \times 10^{7}$ | 2.0667 |
| HCP5 | XXbac-BPG248L24.13 | rs2894207,rs2844502,3 | $1.16 \times 10^{-21}$ | $1.10 \times 10^{6}$ | 2.7467 | rs1960278,rs4947314,4 | $2.87 \times 10^{-18}$ | $2.09 \times 10^{9}$ | 2.1659 |
| CDSN | HLA-C | rs3094205,rs7773175,4 | $1.23 \times 10^{-19}$ | $2.48 \times 10^{6}$ | 2.6222 | rs3094222,rs2524096,5 | $2.83 \times 10^{-18}$ | $9.25 \times 10^{6}$ | 2.5940 |
| HCP5 | RNU6-283P | rs9266490,rs9501106,4 | $1.12 \times 10^{-14}$ | $1.81 \times 10^{6}$ | 2.9946 | rs4358666,rs10223568,5 | $2.52 \times 10^{-19}$ | $4.53 \times 10^{6}$ | 2.0154 |
| HCP5 | MUC22 | rs9394031,rs9501106,4 | $4.48 \times 10^{-16}$ | $1.31 \times 10^{6}$ | 3.4966 | rs13210132,rs45533135,5 | $5.05 \times 10^{-20}$ | $5.71 \times 10^{6}$ | 2.2482 |
| HLA-B | PSORS1C1 | rs1265078,rs2156875,3 | $2.17 \times 10^{-16}$ | 110 613 | 2.3419 | rs6911408,rs2523619,4 | $2.29 \times 10^{-25}$ | $7.73 \times 10^{9}$ | 2.3932 |
| RNU6-283P | XXbac-BPG299F13.15 | rs2844580,rs3130467,2 | $7.02 \times 10^{-20}$ | 4000.37 | 2.5763 | rs1639110,rs4358666,5 | $1.31 \times 10^{-21}$ | $1.60 \times 10^{10}$ | 2.2792 |
| HCG20 | PSORS1C1 | rs12526186,rs1265078,4 | $2.64 \times 10^{-16}$ | 90 558.8 | 2.7948 | rs28894086,rs1265078,4 | $2.99 \times 10^{-17}$ | $4.10 \times 10^{9}$ | 2.0505 |
| PSORS1C1 | RPL3P2 | rs1265078,rs12191877,4 | $6.80 \times 10^{-28}$ | 101 408 | 3.5547 | rs6931633,rs2853935,4 | $7.53 \times 10^{-25}$ | $2.18 \times 10^{9}$ | 2.3316 |
| HCP5 | HLA-C | rs7773175,rs2516510,3 | $1.06 \times 10^{-16}$ | 2864.29 | 2.3592 | rs2853952,rs45533135,5 | $7.64 \times 10^{-29}$ | $5.76 \times 10^{9}$ | 2.4587 |
| CDSN | HCG22 | rs2523857,rs3094205,2 | $1.23 \times 10^{-14}$ | 185 829 | 2.2090 | rs16898629,rs6913137,5 | $2.40 \times 10^{-22}$ | $2.74 \times 10^{6}$ | 2.2284 |
| CDSN | HCP5 | rs3094205,rs9295991,4 | $1.31 \times 10^{-14}$ | 174 387 | 2.3617 | rs6913137,rs45533135,5 | $1.60 \times 10^{-22}$ | $4.11 \times 10^{6}$ | 2.2155 |
| HCP5 | RPL3P2 | rs12191877,rs2844502,3 | $1.35 \times 10^{-27}$ | 51 197.2 | 3.3261 | rs2524052,rs4947314,4 | $4.16 \times 10^{-18}$ | $1.44 \times 10^{9}$ | 2.1490 |
| USP8P1 | XXbac-BPG248L24.13 | rs3132485,rs9380237,2 | $3.64 \times 10^{-15}$ | 153 429 | 2.2475 | rs2844603,rs9468932,4 | $7.15 \times 10^{-24}$ | $1.34 \times 10^{6}$ | 2.3445 |
| HLA-C | LINC01149 | rs7773175,rs2516460,4 | $8.99 \times 10^{-18}$ | 33 819.1 | 2.4525 | rs2524096,rs28575156,4 | $1.51 \times 10^{-20}$ | $4.55 \times 10^{7}$ | 2.3562 |
| HLA-C | USP8P1 | rs3132485,rs7773175,2 | $7.92 \times 10^{-18}$ | 38 353.5 | 2.4264 | rs9264602,rs9468914,5 | $5.92 \times 10^{-15}$ | $1.81 \times 10^{6}$ | 2.3210 |
| PSORS1C1 | WASF5P | rs1265078,rs9468926,3 | $1.36 \times 10^{-14}$ | 1759.12 | 2.2168 | rs6931633,rs2524163,4 | $1.18 \times 10^{-24}$ | $1.39 \times 10^{9}$ | 2.3224 |
| RNU6-283P | XXbac-BPG154L12.4 | rs9266490,rs6916062,4 | $4.25 \times 10^{-16}$ | 27 453.4 | 4.3360 | rs4358666,rs6916062,5 | $2.92 \times 10^{-15}$ | 898 239 | 1.8483 |
| PSORS1C3 | XXbac-BPG248L24.13 | rs887464,rs9380237,2 | $2.97 \times 10^{-14}$ | 18 803.2 | 2.1875 | rs887468,rs9468932,4 | $7.80 \times 10^{-28}$ | $1.29 \times 10^{7}$ | 2.5444 |
| RPL3P2 | ZDHHC20P2 | rs2244027,rs12191877,2 | $4.28 \times 10^{-27}$ | 16 113.6 | 3.2691 | rs12191877,rs13202464,5 | $3.90 \times 10^{-30}$ | $3.10 \times 10^{6}$ | 2.4911 |
| HLA-C | ZDHHC20P2 | rs2244027,rs7773175,2 | $2.35 \times 10^{-15}$ | 129.232 | 2.2599 | rs2853952,rs13202464,5 | $1.52 \times 10^{-28}$ | $2.91 \times 10^{9}$ | 2.4497 |
| CCHCR1 | PSORS1C1 | rs1265078,rs3130453,3 | $3.30 \times 10^{-15}$ | 7258.28 | 2.2521 | rs6929464,rs1265112,4 | $5.22 \times 10^{-16}$ | $8.38 \times 10^{7}$ | 2.0687 |
| HLA-B | HLA-C | rs7773175,rs2156875,3 | $7.53 \times 10^{-17}$ | 4038.26 | 2.3627 | rs2844624,rs2523619,4 | $3.00 \times 10^{-21}$ | $2.16 \times 10^{7}$ | 2.1636 |
| POU5F1 | XXbac-BPG248L24.13 | rs3094188,rs2894207,2 | $8.07 \times 10^{-19}$ | 1585.26 | 2.5253 | rs6929434,rs9380238,5 | $1.55 \times 10^{-17}$ | $1.55 \times 10^{7}$ | 1.9712 |
| HCP5 | XXbac-BPG299F13.15 | rs3130467,rs9501106,4 | $4.43 \times 10^{-19}$ | 634.442 | 3.3513 | rs1639110,rs45533135,5 | $1.28 \times 10^{-19}$ | $1.64 \times 10^{8}$ | 2.0320 |
| XXbac-BPG248L24.13 | ZDHHC20P2 | rs2244027,rs2894207,2 | $2.64 \times 10^{-18}$ | 484.266 | 2.4920 | rs9380237,rs6927154,4 | $7.49 \times 10^{-24}$ | $2.42 \times 10^{8}$ | 2.3400 |
| HLA-C | TCF19 | rs2073717,rs7773175,2 | $3.25 \times 10^{-16}$ | 936.341 | 2.3163 | rs7750641,rs2524096,5 | $3.95 \times 10^{-18}$ | $6.62 \times 10^{6}$ | 2.5844 |
| HCG20 | HLA-C | rs3131039,rs7773175,2 | $9.48 \times 10^{-15}$ | 32.0711 | 2.2230 | rs12527188,rs2853961,4 | $5.51 \times 10^{-19}$ | $7.34 \times 10^{8}$ | 2.3412 |
| XXbac-BPG299F13.15 | ZDHHC20P2 | rs2244027,rs3130517,2 | $5.40 \times 10^{-19}$ | 66.149 | 2.5096 | rs1639110,rs7754026,5 | $3.78 \times 10^{-20}$ | $5.53 \times 10^{8}$ | 2.2293 |
| LINC01149 | PSORS1C1 | rs1265078,rs2516460,4 | $7.68 \times 10^{-14}$ | 311.706 | 2.1767 | rs6931633,rs28575156,4 | $4.52 \times 10^{-23}$ | $3.63 \times 10^{7}$ | 2.6075 |
| DHFRP2 | XXbac-BPG248L24.13 | rs2894207,rs9266329,3 | $5.93 \times 10^{-18}$ | 215.656 | 2.4661 | rs9366778,rs4959062,6 | $1.21 \times 10^{-16}$ | $1.66 \times 10^{7}$ | 4.1400 |
| HLA-C | MICB | rs3131636,rs7773175,4 | $1.46 \times 10^{-15}$ | 208.315 | 2.6027 | rs2524096,rs3130612,5 | $1.79 \times 10^{-17}$ | $1.46 \times 10^{6}$ | 2.4908 |
| HLA-C | RNU6-283P | rs2844580,rs7773175,2 | $5.32 \times 10^{-15}$ | 57.1072 | 2.2327 | rs2853952,rs2523535,3 | $4.78 \times 10^{-27}$ | $9.20 \times 10^{7}$ | 2.3439 |
| C2 | CCHCR1 | rs3130573,rs9267673,4 | $1.79 \times 10^{-20}$ | 119.671 | 3.3632 | rs130069,rs9267673,5 | $3.31 \times 10^{-17}$ | 688 988 | 2.0498 |
| MICA | XXbac-BPG299F13.15 | rs3130467,rs9266844,4 | $2.69 \times 10^{-18}$ | 104.609 | 2.8307 | rs1639110,rs17206680,5 | $8.36 \times 10^{-18}$ | $2.50 \times 10^{6}$ | 1.9661 |
| HLA-C | PSORS1C3 | rs4713438,rs7773175,2 | $9.56 \times 10^{-15}$ | 31.7954 | 2.2270 | rs3130506,rs2249742,2 | $2.35 \times 10^{-16}$ | $3.35 \times 10^{7}$ | 1.9880 |
| C2 | ZDHHC20P2 | rs2442749,rs9267673,2 | $4.05 \times 10^{-20}$ | 52.8453 | 3.0754 | rs13202464,rs9267677,5 | $4.84 \times 10^{-18}$ | $4.99 \times 10^{6}$ | 2.0148 |
| PSORS1C1 | XXbac-BPG299F13.15 | rs1265078,rs3130467,4 | $1.02 \times 10^{-17}$ | 27.5987 | 2.4224 | rs6929464,rs1639110,4 | $8.13 \times 10^{-19}$ | $2.57 \times 10^{7}$ | 2.2778 |
| LINC00243 | XXbac-BPG248L24.13 | rs1264344,rs2894207,2 | $3.54 \times 10^{-17}$ | 36.1766 | 2.4510 | rs4248149,rs9380237,4 | $2.92 \times 10^{-20}$ | $1.39 \times 10^{7}$ | 2.4030 |
| PPIAP9 | RNU6-283P | rs2253907,rs9267464,4 | $2.38 \times 10^{-17}$ | 50.5393 | 3.6310 | rs4358666,rs9267464,5 | $1.22 \times 10^{-15}$ | $2.15 \times 10^{6}$ | 1.8643 |
| HCG22 | HLA-C | rs2523857,rs7773175,2 | $8.42 \times 10^{-15}$ | 36.079 | 2.2320 | rs3131788,rs2524096,5 | $2.66 \times 10^{-18}$ | $9.85 \times 10^{6}$ | 2.5957 |
| HCG20 | XXbac-BPG299F13.15 | rs3129980,rs3130517,2 | $1.01 \times 10^{-18}$ | 35.4352 | 2.5010 | rs28894086,rs3130517,4 | $5.44 \times 10^{-18}$ | $5.53 \times 10^{6}$ | 2.1174 |
| LINC00243 | RPL3P2 | rs886423,rs12191877,2 | $2.61 \times 10^{-24}$ | 26.4495 | 2.9820 | rs12660883,rs2524052,4 | $4.03 \times 10^{-16}$ | $1.22 \times 10^{6}$ | 2.1390 |
| HCP5 | PPIAP9 | rs9501106,rs9267464,4 | $5.23 \times 10^{-17}$ | 22.9963 | 3.9233 | rs45533135,rs9267464,5 | $5.90 \times 10^{-15}$ | $2.04 \times 10^{6}$ | 1.9821 |

**Supplementary Table S8:** This table shows the common gene-gene interactions predicted from the SNP-SNP interactions with top 10000th improvement metric value from datasets phs000019.v1.p1 and phs000982.v1.p1

| Gene | Chr | Start Pos | End Pos | Type |
|---|---|---|---|---|
| LINC00243 | 6 | 30,766,431 | 30,798,436 | Known processed transcript |
| MUC22 | 6 | 30,978,251 | 31,003,179 | Known protein coding |
| HCG22 | 6 | 31,021,227 | 31,027,667 | Known lincRNA |
| PSORS1C1 | 6 | 31,082,527 | 31,107,869 | Known protein coding |
| CDSN | 6 | 31,082,867 | 31,088,223 | Known protein coding |
| CCHCR1 | 6 | 31,110,216 | 31,126,015 | Known protein coding |
| TCF19 | 6 | 31,126,319 | 31,134,936 | Known protein coding |
| POU5F1 | 6 | 31,132,119 | 31,148,508 | Known protein coding |
| PSORS1C3 | 6 | 31,141,512 | 31,145,676 | Known sense intronic |
| HCG27 | 6 | 31,165,537 | 31,171,745 | Known protein coding |
| XXbac-BPG299F13.15 | 6 | 31,190,690 | 31,190,870 | Known pseudogene |
| HLA-C | 6 | 31,236,526 | 31,239,907 | Known protein coding |
| USP8P1 | 6 | 31,243,349 | 31,246,531 | Known pseudogene |
| RPL3P2 | 6 | 31,248,094 | 31,249,296 | Known pseudogene |
| WASF5P | 6 | 31,255,287 | 31,256,741 | Known pseudogene |
| XXbac-BPG248L24.13 | 6 | 31,261,685 | 31,269,419 | Novel lincRNA |
| HLA-B | 6 | 31,321,649 | 31,324,965 | Known protein coding |
| DHFRP2 | 6 | 31,334,129 | 31,334,675 | Known pseudogene |
| RNU6-283P | 6 | 31,337,911 | 31,338,017 | Known snRNA |
| ZDHHC20P2 | 6 | 31,348,188 | 31,348,616 | Known pseudogene |
| HCP5 | 6 | 31,368,479 | 31,445,283 | Known sense overlapping |
| Y_RNA | 6 | 31,369,929 | 31,370,027 | Novel misc RNA |
| MICA | 6 | 31,371,356 | 31,383,092 | Known protein coding |
| LINC01149 | 6 | 31,409,444 | 31,414,750 | Novel lincRNA |
| MICB | 6 | 31,462,658 | 31,478,901 | Known protein coding |
| PPIAP9 | 6 | 31,487,257 | 31,488,068 | Known pseudogene |
| C2 | 6 | 31,865,562 | 31,913,449 | Known protein coding |
| XXbac-BPG154L12.4 | 6 | 32,223,488 | 32,233,615 | Novel antisense |
| HCG20 | 6 | 30,760,027 | 30,760,027 | Known lincRNA |

**Supplementary Table S9:** This table shows the genes found among the 60 common gene-gene interactions predicted from the SNP-SNP interactions with top 10000 improvement metric value from datasets phs000019.v1.p1 and phs000982.v1.p1

| No. of SNP | Mode | Heritability | | | |
|---|---|---|---|---|---|
| | | 0.1 | 0.2 | 0.3 | 0.4 |
| 1000 | Without Look-up Table | 54.81s | 54.93s | 54.82s | 54.97s |
| | With Look-up Table | 6.155s | 6.303s | 6.280s | 6.292s |
| | Speed-Up | 8.906x | 8.715x | 8.729x | 8.737x |
| 5000 | Without Look-up Table | 1470s | 1467s | 1465s | 1469s |
| | With Look-up Table | 138.2s | 138.3s | 138.366s | 138.5s |
| | Speed-Up | 10.63x | 10.61x | 10.59x | 10.60x |
| 10000 | Without Look-up Table | 5777s | 5784s | 5783s | 5833s |
| | With Look-up Table | 550s | 551s | 551s | 551s |
| | Speed-Up | 10.50x | 10.50x | 10.50x | 10.59x |

**Supplementary Table S10:** This table shows the runtime of the program with or without the pre-computed look-up table under different datasets.

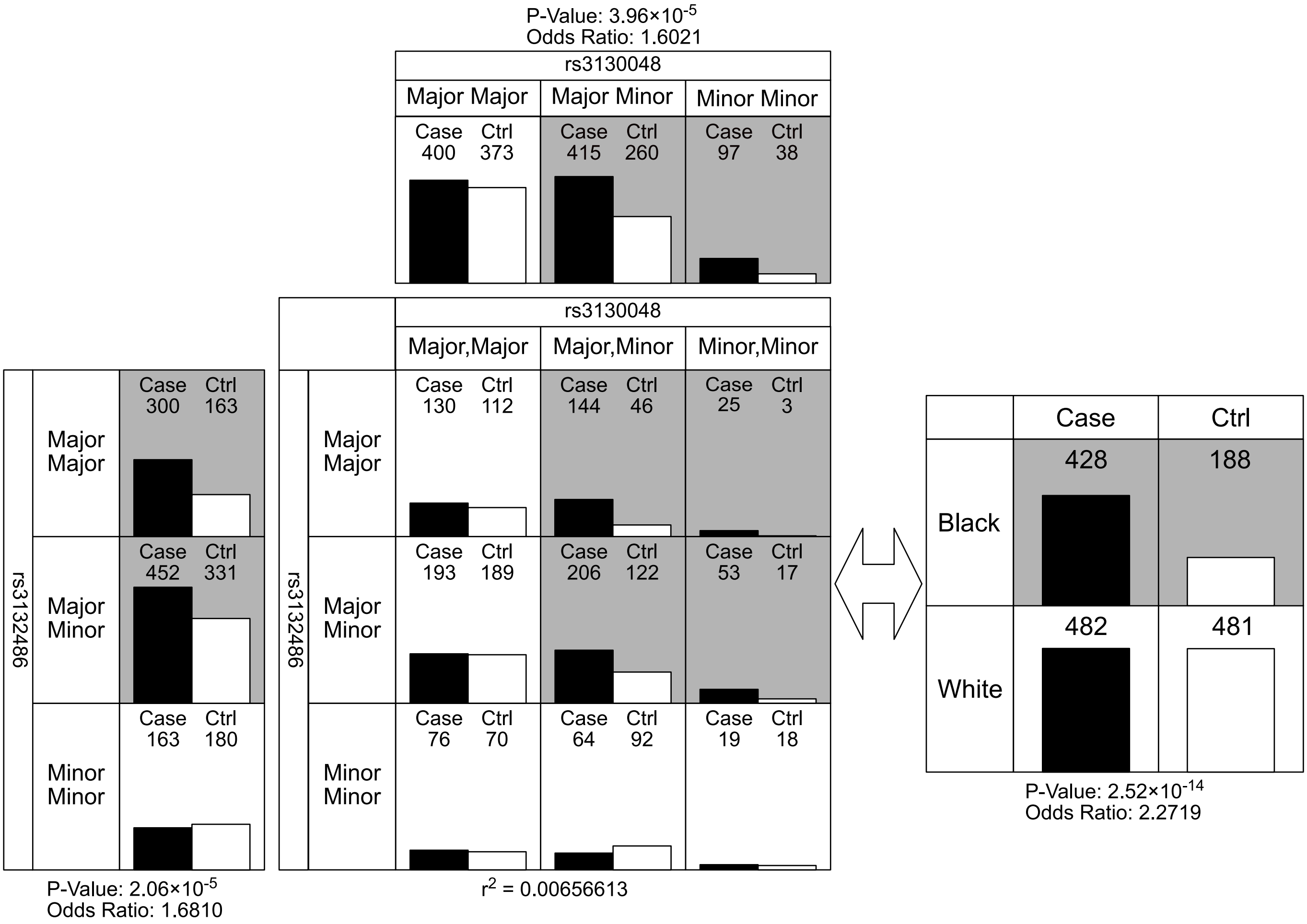


**Supplementary Figure S1:** This figure shows the interaction between SNPs rs3132486 and rs3130048 found from dataset phs000019.v1.p1 which is an example of an potential non-linear SNP-SNP interaction.

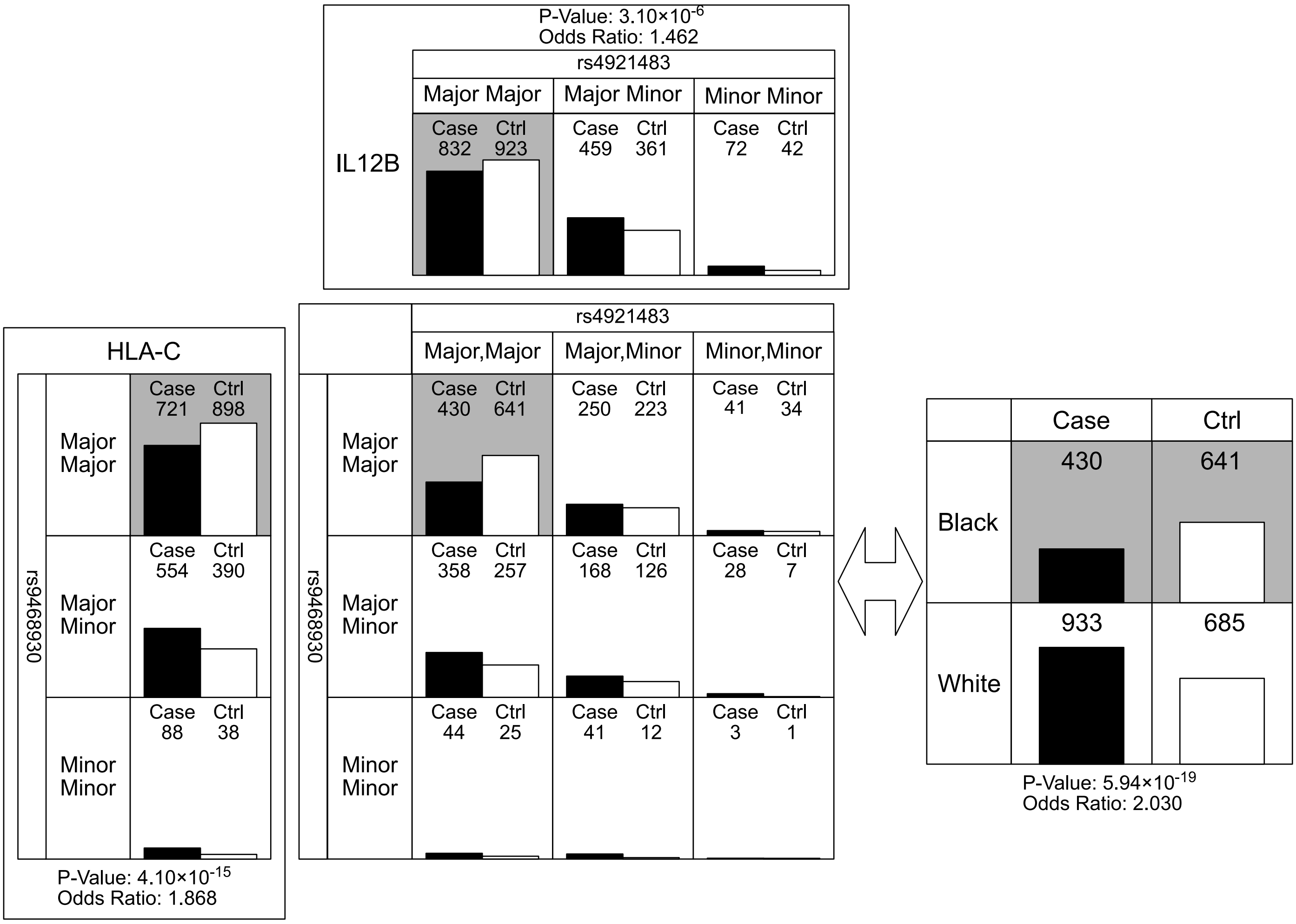


**Supplementary Figure S2:** This figure shows the interaction between two SNPs rs9468930 and rs4921483 imputed from dataset phs000982.v1.p1. which is an example of a SNP-SNP interaction between two psoriasis associated genes *HLA-C* and *IL12B*.

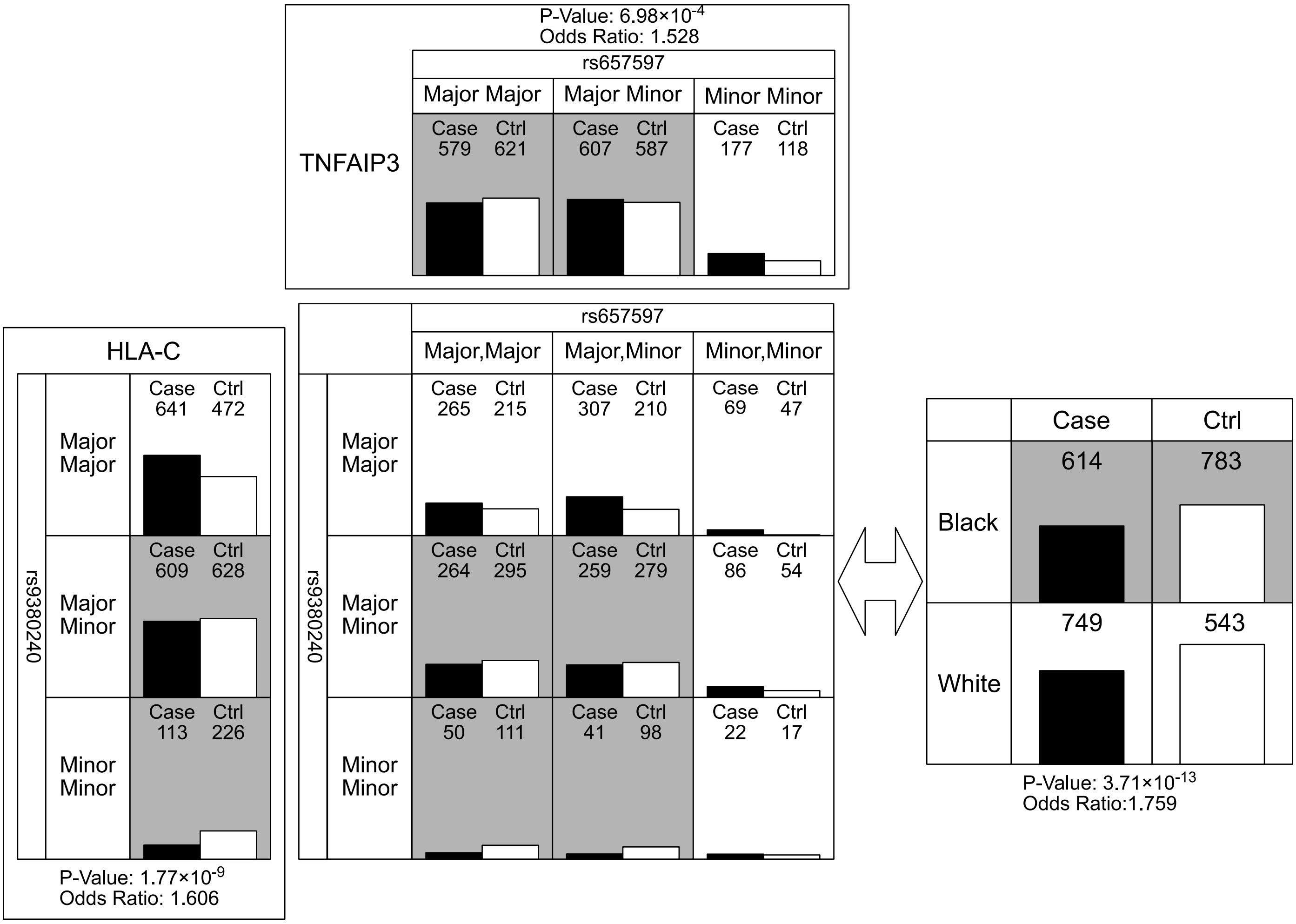


**Supplementary Figure S3:** This figure shows the interaction between two SNPs rs9380240 and rs657597 imputed from dataset phs000982.v1.p1 which is an example of a SNP-SNP interaction between two psoriasis associated genes *HLA-C* and *TNFAIP3*.

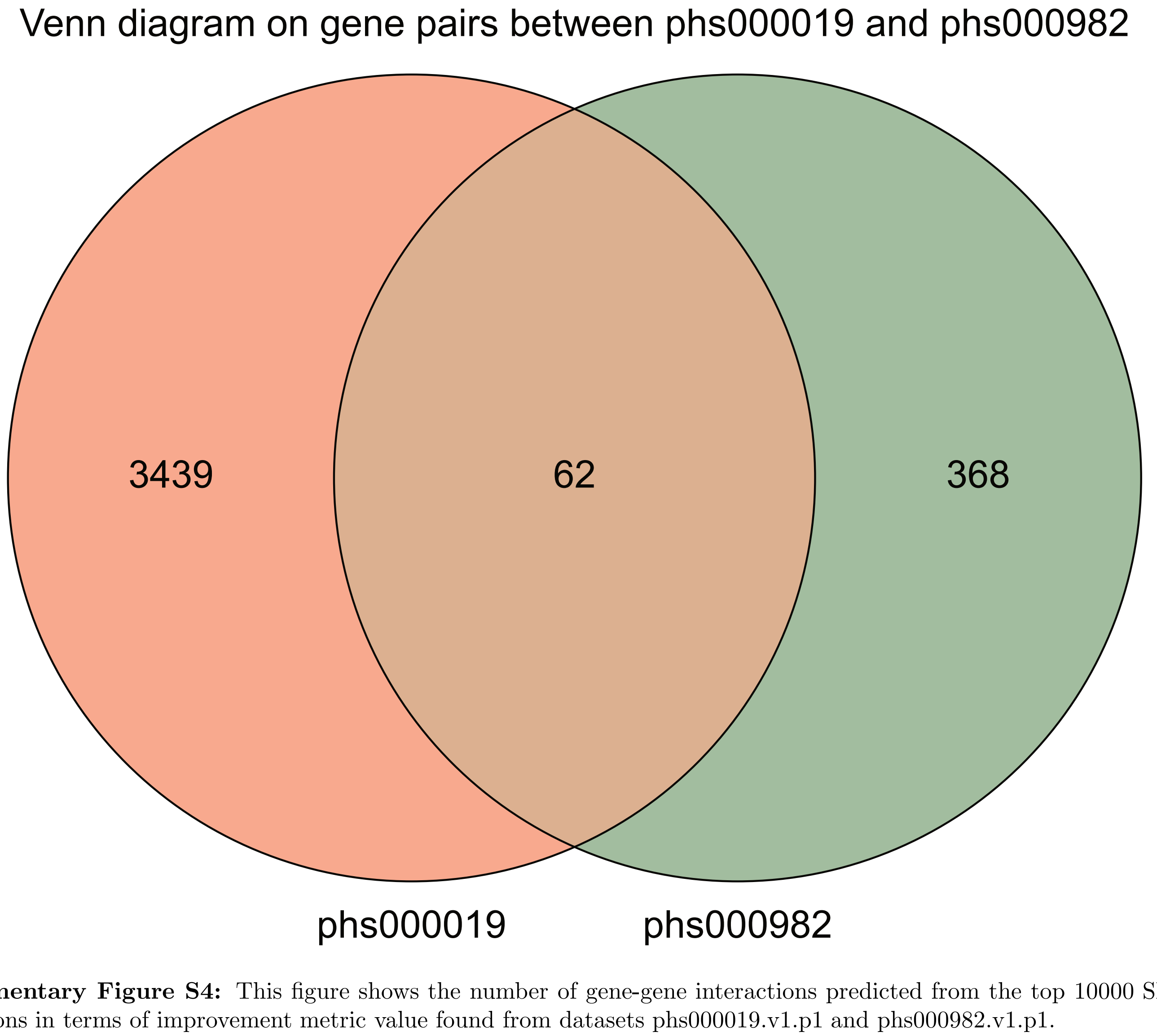


**Supplementary Figure S4:** This figure shows the number of gene-gene interactions predicted from the top 10000 SNP-SNP interactions in terms of improvement metric value found from datasets phs000019.v1.p1 and phs000982.v1.p1.

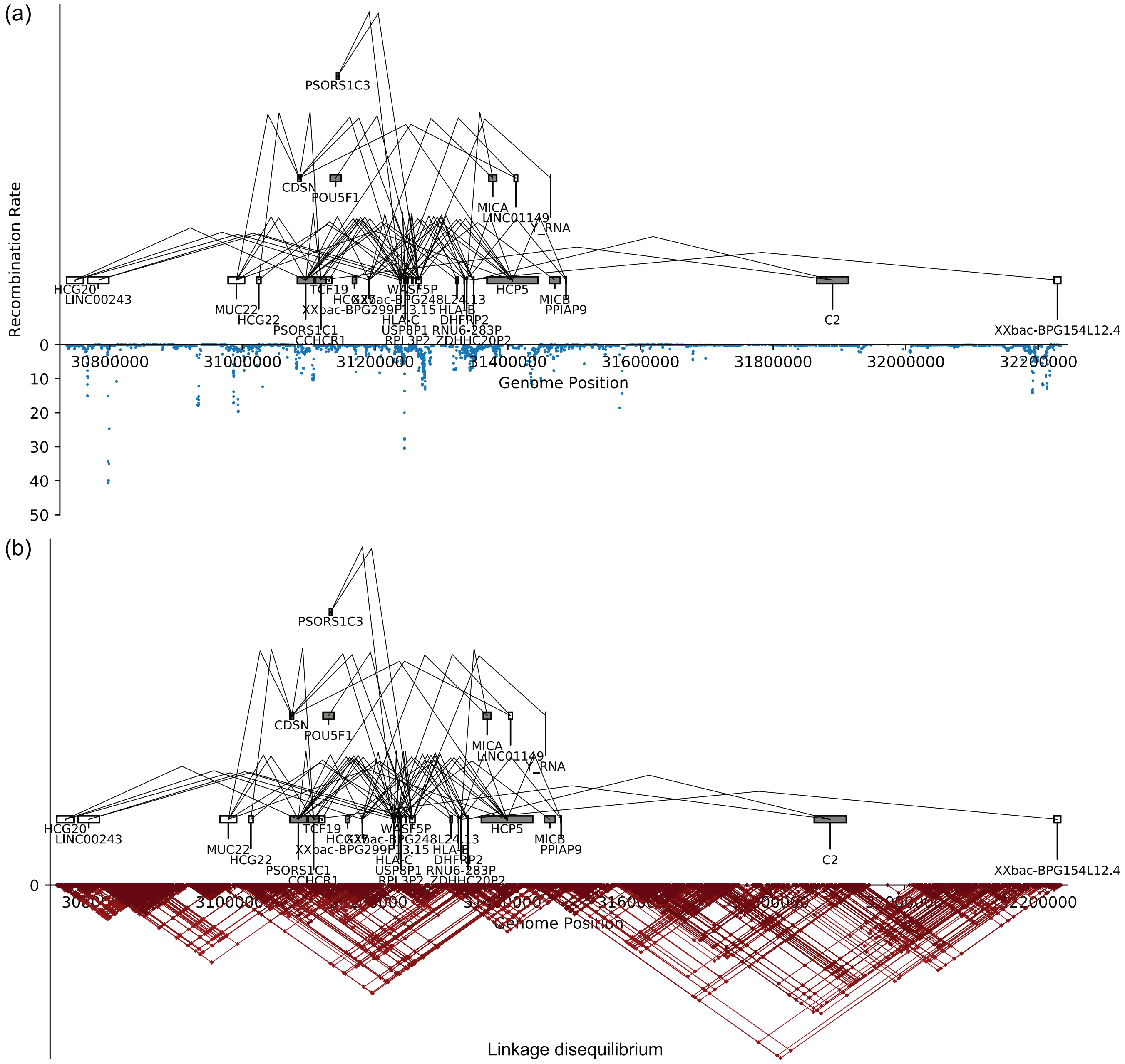


**Supplementary Figure S5:** In this figure, both parts (a) and (b) show the gene network constructed from 60 common gene-gene interactions predicted from the top 10000 SNP-SNP interactions in terms of improvement metric value found from datasets phs000019.v1.p1 and phs000982.v1.p1 with a linear layout, where the genes are laid down according to their genomic position in Chromosome 6. The genomic combination rate and the SNP pairs with a significant linkage disequilibrium score (r larger than 0.9) along Chromosome 6 from position 30734602 to 32233615 are plotted in parts (a) and (b) respectively.

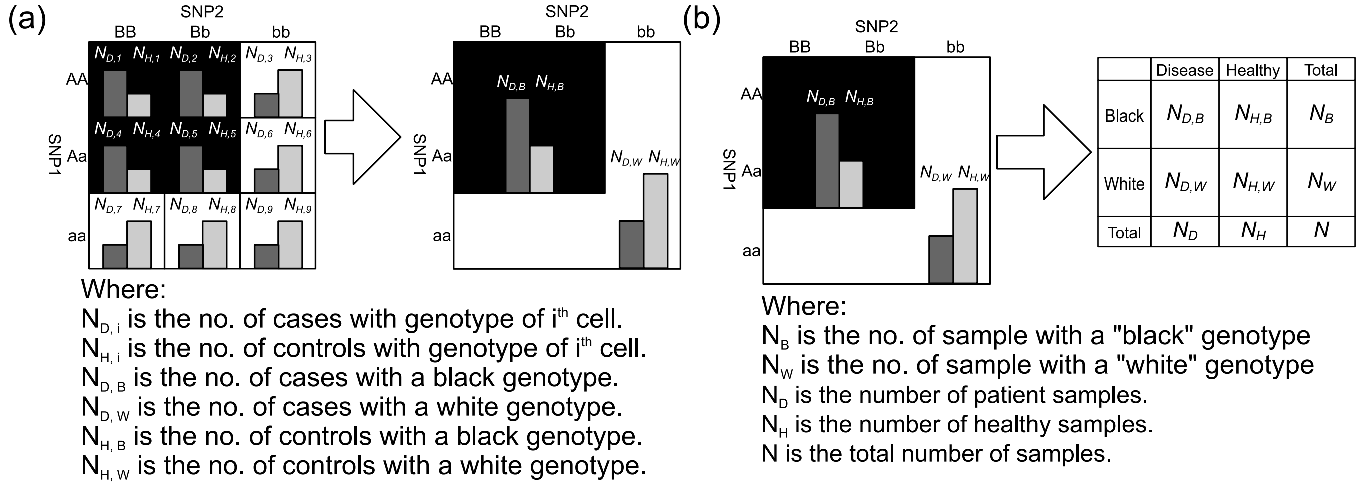


**Supplementary Figure S6:** In this figure, part (a) shows the process of aggregating the number of cases and controls with black and white genotypes in the 3x3 genotype table of $SNP_1$ and $SNP_2$, where the genotypes are coloured according to the pattern 1 in Fig. 5b. Meanwhile, part (b) shows the process of arranging the total numbers of cases and controls calculated in part (a) into a 2x2 contingency table. Major alleles are represented by upper-case letters (i.e. A, B) and minor alleles are represented by lower-case letters (i.e. a, b).
